# Evolution mechanism and progressive reshaping model of driving behaviors when humans take over intelligent vehicles

**DOI:** 10.1038/s44172-025-00510-6

**Published:** 2025-10-31

**Authors:** Ziyu Zhang, Chunyan Wang, Zhongkai Luan, Wanzhong Zhao

**Affiliations:** College of Energy and Power Engineering, Nanjing University of Aeronautics and Astronautics, Nanjing, China

**Keywords:** Mechanical engineering, Science, technology and society

## Abstract

Despite the rapid development of autonomous driving, drivers still need to take over when autonomous driving exceeds its design scope or malfunctions. The role of drivers is undergoing a significant transformation from operators to backup users. The existing human driving behaviour models focus more on the behaviour of humans as operators, with static and time-invariant characteristics. However, as backup users, human behaviour characteristics during the takeover process exhibit dynamic and time-varying characteristics, and traditional driver models can no longer describe these, making it difficult to support the safe development of autonomous driving. Unfortunately, the evolution mechanism of driver behaviour is unclear, which has led to the continuous occurrence of accidents in autonomous vehicles. To support the safe development of autonomous driving, we studied the changes in drivers’ cognition, decision-making, and control behaviours during the takeover, revealing the evolution mechanism of driver behaviours during the takeover. On this basis, a progressive reshaping model of human driving behaviours is constructed. The comparison with actual driver control data shows that the accuracy of the proposed model is 88.57%, providing a new perspective for understanding driver behaviour during emergency takeover and having certain application value in the research of autonomous driving technology.

## Abstract (web summary)

Ziyu Zhang and colleagues propose a progressive reshaping model of human driving behaviour. It provides a new perspective for understanding driver behaviour during takeover from autonomous vehicles, guiding development of more secure autonomous driving systems.

## Introduction

Autonomous driving technology plays a vital role in human society. In the past few years, it has made major breakthroughs and has great potential for development in the future^1–3^. However, in some special scenarios, such as beyond the design scope or system failure, human drivers are still needed as backup users to takeover the vehicle driving^4–6^. However, the behaviors of humans in the takeover process are very different from those of traditional vehicle driving, which makes autonomous driving accidents happen frequently. In order to realize the safe development of autonomous driving technology, it is necessary to study the driver’s behavior and build a behavior model.

For drivers, their driving behaviors include cognition, decision-making, and control^7–9^. McRuer et al. proposed the Crossover driver model, which can describe the functions of humans as compensation, tracking, and prediction^10^. However, it lacks the description of driver behavior at high speed. So, Guo et al. proposed the driver preview-following theory, which reflects the driver’s cognitive and reaction behaviors^11^. Subsequently, Salvucci et al. proposed the driver’s two-point preview mechanism, believing that the driver makes steering decisions through this behavior^12^. Wang et al. built a two-point preview model of the driver, which has a better description of the driver’s preview behaviors^13,14^^.^ For the driver’s decision-making behavior, Lanei et al. used the linear time-varying model prediction to construct the driver’s steering decision behavior^15^^.^ Chen et al. proposed a compensatory driver control model based on fuzzy control^16^. Axenie et al. combined fuzzy logic to produce a reasonable driver steering decision model^17^. With the development of deep learning theory, neural network models with excellent approximation ability have been gradually applied to the modeling of drivers’ steering decision behavior^18^. Hassan et al. used dynamic artificial neural networks based on natural driving data to model the driver’s steering behavior^19^. Chong et al. proposed a rule-based neural network model for simulating the longitudinal and lateral decision behavior of drivers^20^^.^ For the driver’s control behavior, Pick et al. built a neuromuscular dynamic model that can represent the driver’s steering control action, which is now widely used^21^. In addition, Lazcano et al. built a comprehensive driver model and realized the comprehensive characterization of the driver’s steering control behavior^22,23^.

The research of the above driver model is mainly used to describe the driving behavior of the driver as the operator. With the improvement of the vehicle's intelligence level, the vehicle gradually has the ability to drive autonomously, and the driver gradually exits the main driving task and only takes over the vehicle in emergency situations. In order to study the changes of human driving behaviors in the takeover scenario, researchers analyzed the changes of visual and muscle characteristics of drivers in the takeover task and proved that drivers have characteristics of driving state changes during the takeover process^24–28^. Wachirawit et al. conducted an emergency takeover experiment using eye tracking, heart rate monitoring, and operation data to observe the driver’s state. The results showed that the response time of the driver’s takeover varied depending on different driving scenarios, and the takeover environment had a differential impact on the driver’s behaviors. The driver’s behavior had different changing characteristics under different takeover scenarios^29^. Xu et al. conducted a series of driving simulator takeover experiments on 12 participants, comparing takeover steering and traditional manual steering scenarios using neuromuscular signals and driver control data. The results showed that there was a gradual recovery of driver behavior changes during the takeover process^30^. Avinoam et al. conducted an emergency takeover experiment with two participants, and the results showed that drivers’ takeover strategies and behavioral choices varied according to the driving order arranged in chronological order^31^.

In summary, current research on driver behavior characteristics focuses more on traditional manual driving scenes, and there is no systematic conclusion and unified theoretical system on the changes in driver behavior characteristics in emergency takeover scenes. Although research on the characteristics of driver takeover behavior has shown its time-varying nature, we still do not understand the evolutionary mechanism of human driver behavior during the takeover process, which cannot support the construction of driver time-varying behavior models in emergency takeover scenarios during high-level autonomous driving stages. Meanwhile, existing steady-state and static driver behavior models for conventional manual driving scenarios cannot describe the time-varying characteristics of driver behavior during emergency takeover processes, and cannot meet the development needs of emergency takeover control strategies for high-level autonomous driving stages. Therefore, it is urgent to clarify the behavioral evolution mechanism of human drivers when taking over intelligent vehicles, and construct a driver behavior model that can reflect their behavioral evolution characteristics to support the safe development of autonomous driving technology.

**Fig. 1:**
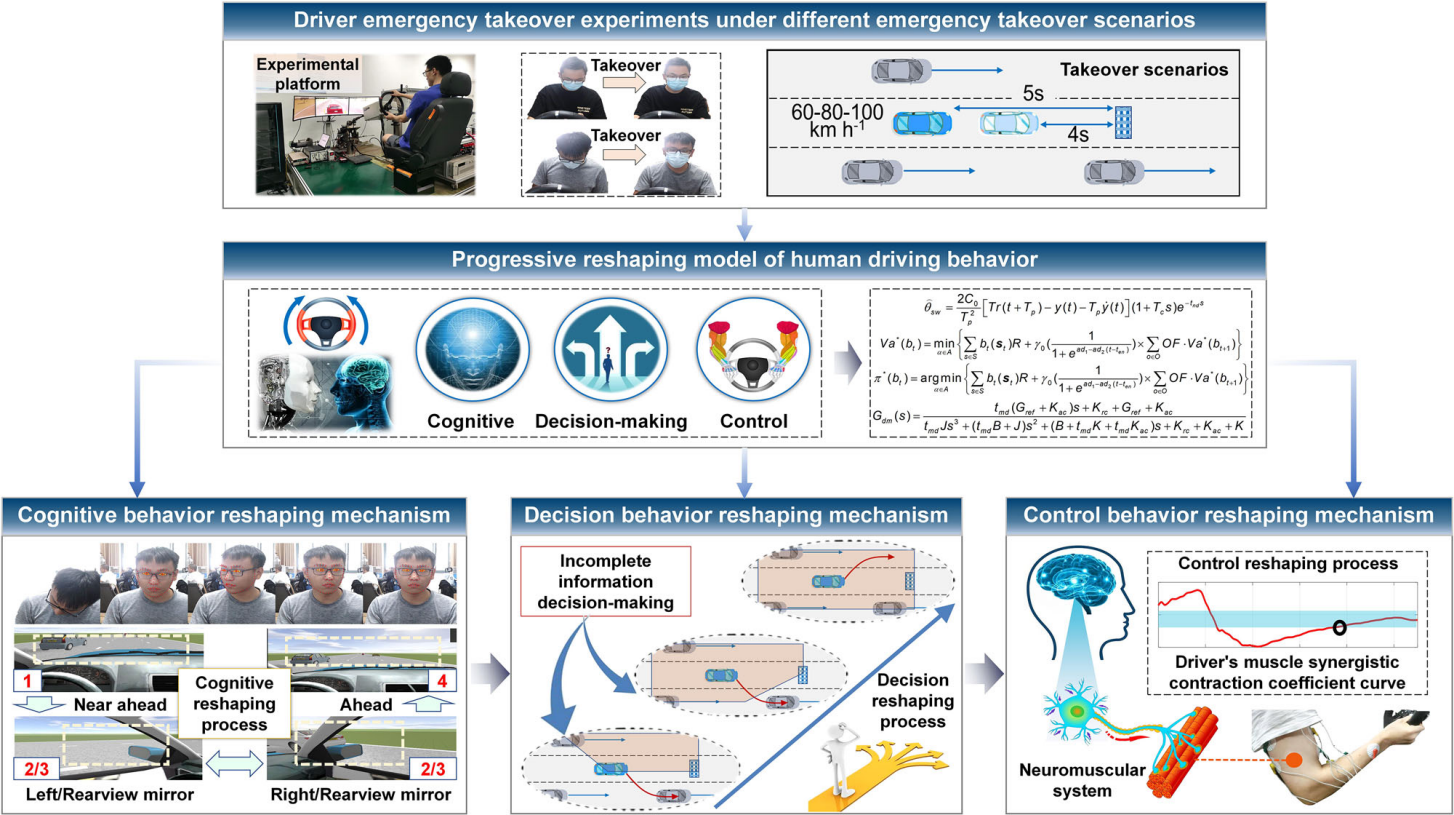
Evolution mechanism and progressive reshaping model of driving behavior when humans takeover intelligent vehicles.

In this paper, based on the hardware-in-loop experimental platform, we organize and carry out an autonomous vehicle takeover experiment. The experimental participants include human drivers with different driving proficiency and gender, and the experimental scenarios include different speeds and reserved takeover time. By recording the visual and myoelectric signals of human drivers and vehicle status signals, we analyze in detail the changes of human driver’s cognition, decision-making and control behavior during the takeover process, reveal the evolution mechanism of human driving behavior during the takeover process, and propose a driver behaviors progressive reshaping model that can describe the time-varying behavior characteristics of human takeover process, as shown in Fig. 1.

The results show that compared with the existing human driver behavior models, the proposed driver behavior model can fully describe the time-varying behavior characteristics of human drivers. Compared to existing research, the main contributions of this article are as follows:

(1) This work no longer focuses on manually driven vehicles like traditional research, but on the impact of driver role changes on their driving behavior under the irreversible trend of vehicle intelligence. It first discovers the progressive reshaping of different behaviors of drivers during the takeover process, and conducts a systematic and comprehensive analysis and research on the changes in drivers’ emergency takeover behavior characteristics. The causes, processes, and adverse effects of driver behavior changes are described in detail.

(2) This work innovatively proposes a method for observing the cognitive, decision-making, and control behavior characteristics of drivers based on their visual scanning, staring, and preview behaviors, as well as the degree of muscle coordination contraction. By utilizing the strong correlation between drivers’ physiological characteristics and their behavioral changes, a time-domain joint analysis of visual behavior and analysis of the collaborative activation characteristics of different muscle groups are adopted to further reveal the evolution mechanism of drivers’ different behaviors.

(3) This work is the first to mathematically describe and express the behavioral changes of drivers during emergency takeover processes. Unlike existing fixed and static driver behavior models for conventional manual driving scenarios, the proposed driver behavior progressive reshaping model is a type of dynamic model that can effectively describe the time-varying reshaping characteristics of driver behavior during takeover processes, thus filling the current gap in research on driver behavior modeling in emergency takeover scenarios. It is a supplement and innovation to the existing theoretical system of driver behavior modeling.

To observe the characteristics of emergency takeover behavior of drivers and verify the superiority and rationality of the proposed driver model, we built an emergency takeover hardware-in-the-loop test platform and organized 15 drivers with different driving proficiency and gender to participate in the takeover experiments. The results indicate that drivers exhibit a progressive reshaping behavior during emergency takeover, and there is a temporal cross-correlation between different behaviors. In addition, we compared and analyzed the driving data of real drivers with the driving data of the model, verifying the feasibility and rationality of the proposed model. Then, we conducted qualitative and quantitative comparative analysis between the proposed model and the existing driver model. The results showed that the proposed model has greater advantages in driver behavior representation and application scenarios, with an accuracy of 88.57% in representing driver emergency takeover behavior.

## Results

Based on the emergency takeover experiment data, we found that the cognitive, decision-making, and control behavior characteristics of drivers during the emergency takeover process exhibit a progressive reshaping characteristic that gradually recovers over time. That is, the behavior characteristics of drivers during the takeover process show a gradual recovery process from a low-level state to a high-level state. Meanwhile, the progressive reshaping process of driving behavior varies among different drivers, meaning that the patterns of changes in the recovery of different driving behaviors over time differ. In addition, there are significant differences in the progressive reshaping time of different drivers’ behaviors; that is, the interval time between the process of drivers recovering from a low-level state to a normal high-level state is different. For this, we analyze the different behavioral characteristics of drivers, reveal the progressive reshaping mechanism of their cognition, decision-making, and control behavior, construct a progressive reshaping model of their behavior and conduct experimental verification.

### Driving behaviors are progressively reshaping the characteristics of human drivers in the takeover process

**Fig. 2:**
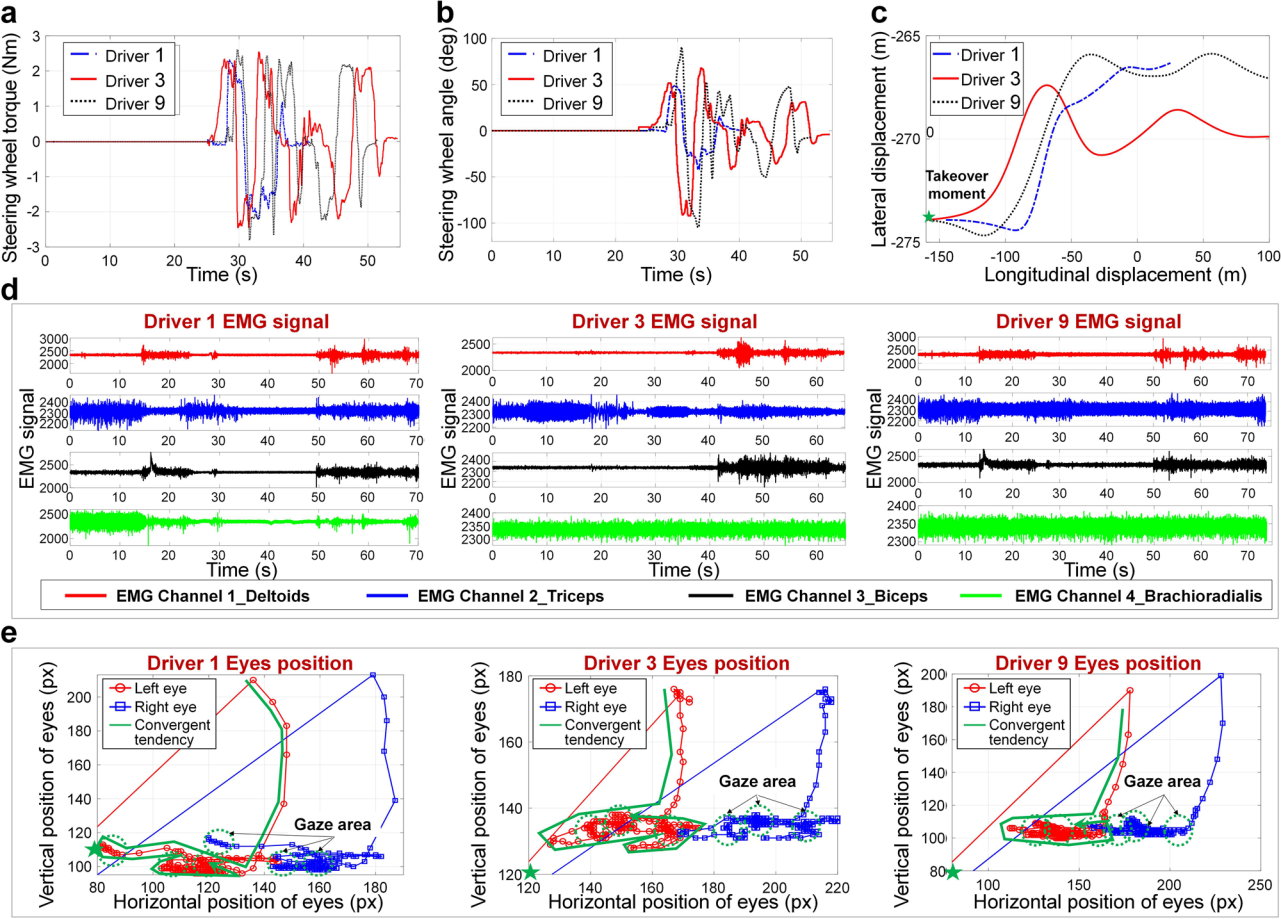
Driving behavior data of different drivers during the takeover process. **a** Steering wheel torque for different drivers. **b** Steering wheel angle for different drivers. **c** Vehicle lateral and longitudinal displacement during the takeover process by different drivers. **d** EMG signal of different drivers (EMG signal is a signal for recording the electrical activity produced by driver’s skeletal muscles). **e** Eyes position of different drivers.

To analyze the characteristics of driver takeover behavior, this paper divides drivers into novice drivers, general drivers, and skilled drivers based on their driving experience. And randomly select one novice, one general, and one skilled driver as cases, and qualitatively analyze their vehicle trajectory, steering angle, electromyogram (EMG), and eye movement signal during the experiment, as shown in Fig. 2.

According to Fig. 2a,b and Supplementary Tab. 1, it can be seen that the process from the driver receiving the takeover instruction to the completion of the steering obstacle avoidance task can be divided into three stages, namely the no-control stage, the overshoot stage and the stable stage, reflecting the process of the driver’s state from offline to unstable and then to stable. This characteristic can be demonstrated by the standard deviation variation of driver control data in Supplementary Tab. 1. The steering wheel angle variation of different drivers gradually increases from 0–3 s, reaches its peak at 3–6 s, and then gradually decreases after 6-9 s. But according to Fig. 2a–c, it can be clearly seen that during the initial 0–1 s of takeover, the driver has almost no steering control. Therefore, the non-control stage of the vehicle is usually between 0–1 s, the overshoot control stage is between 2–6 s, and the stable stage is after 6 s.

According to Fig. 2d, e, it can be seen that eye movement signals of different drivers present a progressive convergence characteristic during the driver takeover process, and the EMG signals of the driver’s arm muscles also show a progressive and orderly characteristic from full activation to ordered cooperative activation. Since the driver’s cognitive behavior is mainly manifested in eye movement, and its control characteristics are mainly determined by its muscle characteristics, this indicates that the driver’s cognitive and control behaviors show progressive reshaping characteristics during the takeover process.

Moreover, it can also be seen from Fig. 2e that the driver’s sight points present a state from sparse edge to dense middle, that is, after receiving the takeover instruction, the driver first scans the environment, and then gazes at a specific area after understanding the environmental status information. At the same time, according to the temporal relationship between the eye information and the steering information, it can be seen that after the driver produces a gaze action, they will perform steering control. It can be considered that the driver’s gaze behavior towards the road is a reference for forming their control decisions. In addition, according to Fig. 1 and Supplementary Tab. 1, it can be seen that the standard deviation of steering wheel angle and lateral displacement of different drivers exhibits a gradually stable characteristic of increasing first and then decreasing with the takeover time, indicating that the driver’s steering behavior and vehicle trajectory also exhibit a gradually stable characteristic. At the same time, different drivers exhibit multiple staring behaviors during the takeover process, indicating that the drivers make multiple decisions during the takeover process. Based on the comparison of their steering data, it can be seen that the later decisions are more stable than the earlier ones, indicating that the driver’s decision behavior also exhibits a progressive reshaping characteristic. Meanwhile, based on the data curves of different drivers, it is not difficult to find that there are certain differences and behavioral intersections in the cognitive reshaping behavior, decision-making reshaping behavior, and manipulation reshaping behavior of different drivers.

### Cognitive behavior progressive reshaping mechanism of human drivers

**Fig. 3:**
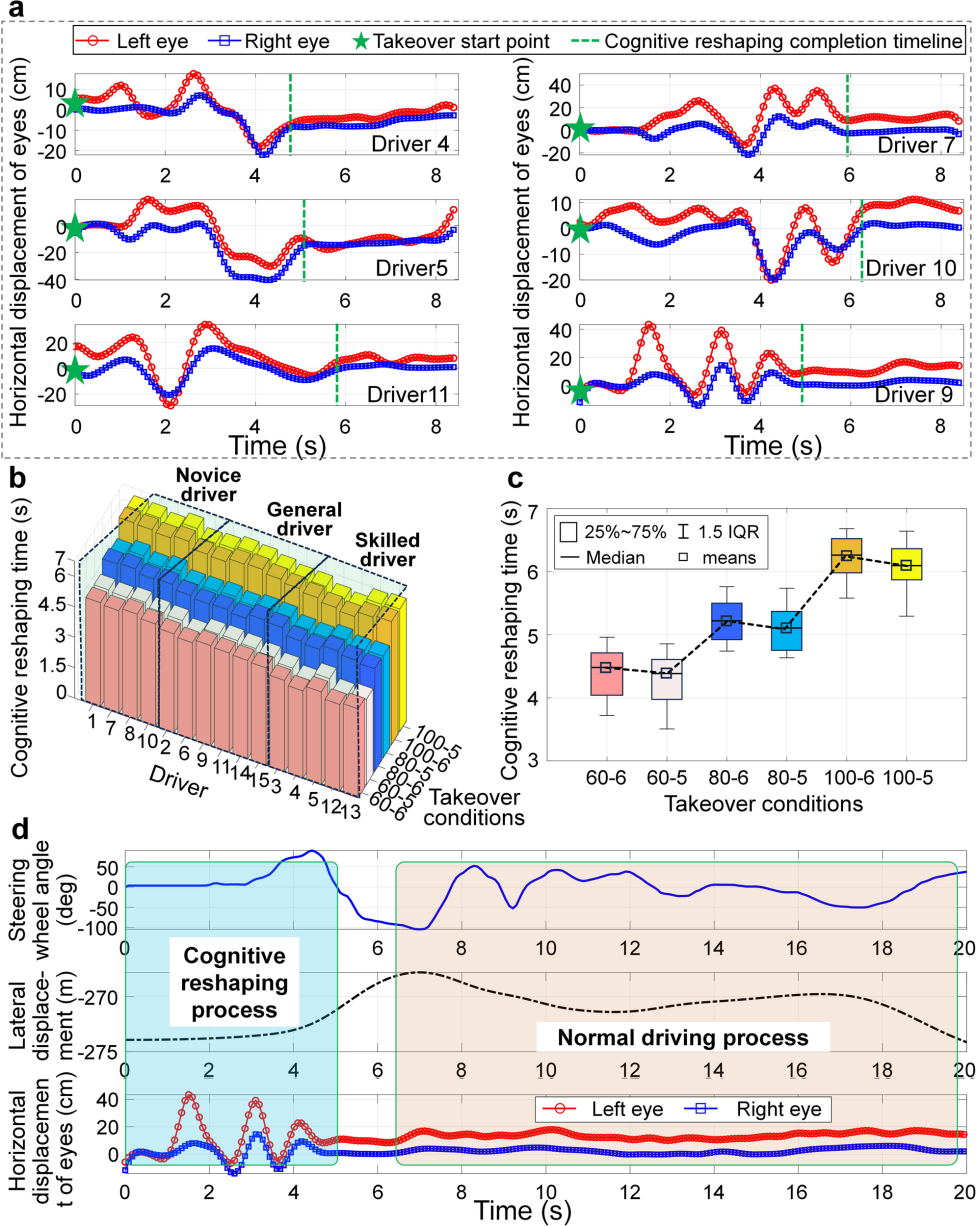
Comparison of eye movement signals and cognitive reshaping time for different drivers. **a** Horizontal displacement of the eyes for different drivers. **b** 3-D histogram of cognitive reshaping time for different drivers. **c** Boxplot of cognitive reshaping time for different drivers. **d** The timing curve of driver takeover status.

During the takeover process, human cognitive behavior is mainly aimed at reacquiring the perception of road risks, that is, reshaping their understanding of the current environment. Usually, the cognitive activities of drivers are accompanied by eye movement, such as scanning the rearview mirror to understand the risk of the target lane before changing lanes during normal manual driving^32^. Therefore, in order to further analyze the cognitive behavior of drivers, we extract horizontal displacement data of the eyes from 2 novice drivers, 2 regular drivers, and 2 skilled drivers as examples for analysis, as shown in Fig. 3a. The five-pointed star in the figure marks the point where the takeover command is issued, and the green dashed line represents the timeline of the completion of the driver’s cognitive reshaping (the time point when the driver produces a gaze action ahead after observing the environment).

From Fig. 3a, it can be seen that different drivers will scan the environment during the initial stage of takeover after receiving the takeover command. This is manifested in the continuous oscillation of the horizontal displacement of the driver’s eyes in the scene. During this process, the driver observes the left and right rearview mirrors and the road ahead to quickly understand the current driving situation. However, according to the green dashed line markings in the figure, it can be seen that there are certain differences in the cognitive reshaping process among different drivers, mainly reflected in the varying length of cognitive reshaping time among different drivers, which is mainly influenced by their driving proficiency, takeover speed, and reserved takeover time.

To further analyze the influence of different drivers’ proficiency, takeover speed, and reserved takeover time on drivers’ cognitive behavior, we extract the cognitive reshaping time and corresponding driver characteristics and working conditions of all drivers, and draw a 3-D histogram and boxplot for quantitative comparison, as shown in Fig. 3b, c. In addition, in order to clarify the impact of driving proficiency on cognitive reshaping time, we analyze drivers with different driving proficiency levels. At the same time, for the sake of simplicity in drawing, the vehicle speed and reserved takeover time in the takeover condition are combined, and the units are omitted to form the coordinate axis.

According to Fig. 3b, c and Supplementary Tab. 2, it can be seen that in terms of driver proficiency, the cognitive reshaping time of skilled drivers is faster than that of novice drivers in all driving conditions. It indicates that the experience of drivers in their driving career is beneficial for them to quickly understand environmental risks and form cognition. In terms of the vehicle speed, according to the boxplot, it can be seen that as the vehicle speed increases during the takeover, different drivers show a characteristic of increasing cognitive reshaping time. It indicates that when drivers takeover the vehicle at a faster speed, the tension caused by the high speed makes them less cautious about the environment. In terms of reserved takeover time, according to the boxplot, it can be seen that the cognitive reshaping time of drivers at different speeds decreases to some extent after the reserved time is shortened. This is because in more urgent situations, drivers make quick decisions for safety reasons, but their understanding of the environment may not be comprehensive. In addition, to simplify the notation, we concatenate the vehicle speed and the reserved takeover time in the takeover conditions. For example, 60-6 in Fig. 3 and 60 km h^−1^_6s in Supplementary Tab. 2 both represent the takeover condition of a vehicle speed of 60 km h^−1^ with a reserved takeover time of 6 s.

To further clarify the cognitive reshaping process of the driver’s driving task in the early stage of takeover, we further compare and analyze the driver’s eye movement information, driver control information, and vehicle trajectory. Figure 3d shows the takeover state curve of the driver at a speed of 80 km h^−1^ and a reserved takeover time of 6 s.

The green dashed box in Fig. 3d represents the cognitive reshaping process of the driver in the early stage of takeover. Combining the driver’s steering angle and vehicle trajectory, it can be seen that in the early stage of this stage, the driver did not manipulate the vehicle, only scanned the environment with their eyes to obtain the status. In the latter half of this stage, the driver made a certain area of gaze movement, which is reflected in the figure where the continuous points gradually stabilized. Meanwhile, in the second half of the stage, the driver has some steering control input, but the vehicle has not produced too much lateral displacement at this time. It can be considered that the driver has made the expected lane selection and started to perform exploratory control actions.

In summary, it can be found that the main tasks of drivers in the cognitive reshaping stage include two aspects: one is to recognize environmental risks, and the other is to make preliminary decisions on the expected lane. This indicates that preliminary decisions will be made due to overly urgent environments before drivers have formed sufficient cognition.

### Decision behavior progressive reshaping mechanism of human drivers

The decision behavior of human drivers is mainly based on the perception of environmental risk information to make judgments in the current scenario, and then determine the ideal trajectory and steering action that can ensure the safety of vehicles driving^33^. According to Fig. 3d and human cognitive psychology, it can be seen that drivers will generate expected lane goals in the second half of cognitive reshaping, which actually reflects the decision-making behavior that exists in the driver’s cognitive process of environmental formation. Therefore, it can be considered that there is a temporal overlap between the cognitive behavior of drivers and their decision-making behavior, which is also in line with the actual behavior characteristics of drivers observing road conditions while driving. However, under the condition of only the target lane, it is difficult for the driver to form continuous and stable steering actions^17,19,33^. Therefore, the driver’s decision-making task is to determine the target trajectory to be tracked and the steering instructions to be executed based on cognition to avoid obstacles.

**Fig. 4:**
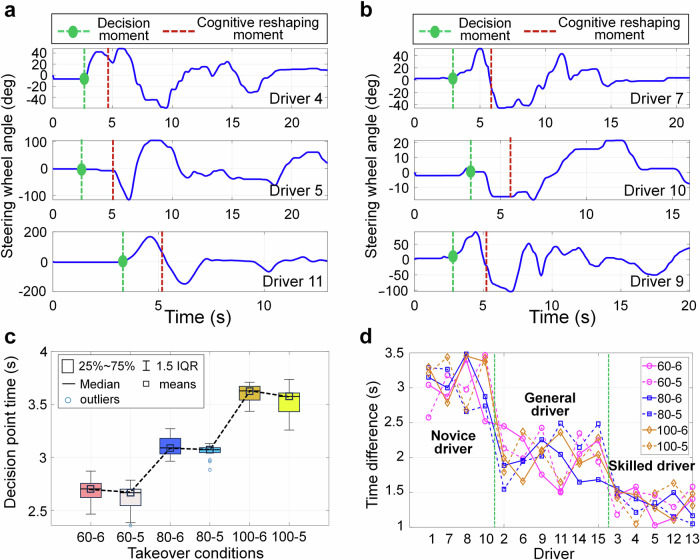
Steering wheel angle and trajectory decision points of different drivers. **a** Steering wheel angle of drivers 4, 5, and 8. **b** Steering wheel angle of drivers 7, 9, and 10. **c** Comparison of decision point time for different drivers. **d** Time difference between decision point time and cognitive reshaping time for different drivers.

To analyze the driver’s decision-making behavior, we define the starting point of the steering action as the trajectory decision point, that is, the driver begins to make intentional decisions about the trajectory target to be tracked, and select data from two novice drivers, two general drivers, and two skilled drivers as examples for analysis, as shown in Fig. 4a, b. The red dashed line in the figure represents the cognitive reshaping timeline, and the green dashed line represents the trajectory decision point. According to the decision point and cognitive reshaping timeline position in the figure, it can be found that the trajectory decision points of different drivers are all earlier than the cognitive reshaping timeline, indicating that drivers will make decisions in advance before cognitive reshaping is completed to avoid collision risks.

To further analyze the differences in decision-making characteristics of different drivers under different working conditions and reserved time, we extract the decision point time and corresponding driver characteristics and working conditions of all drivers, and draw a boxplot for quantitative comparison, as shown in Fig. 4c. In addition, to clarify the relationship between decision point time and cognitive reshaping time, we compare the decision point time and cognitive reshaping time of different drivers, as shown in Fig. 4d. According to Fig. 4c and Supplementary Tab. 3, the driver’s decision point time and cognitive reshaping time have similar characteristics, which may be because the driver only makes decisions after obtaining certain environmental cognition, and therefore the decision point time is influenced by the environmental cognitive reshaping time. According to Fig. 4d, the difference between the decision point time and cognitive reshaping time of different drivers is less affected by operating conditions. Drivers of the same type exhibit similar characteristics under different operating conditions, while drivers with different driving proficiency show significant differences. Since the driver has already produced clear steering and gaze actions after cognitive reshaping is completed, it can be considered that the time point at which cognitive reshaping is completed is the moment when trajectory decision-making is completed. Therefore, the time difference between the two can be regarded as the driver’s decision-making time, that is, the trajectory decision-making reshaping time. According to the decision time difference of different drivers, it can be seen that the trajectory decision time and cognitive reshaping time difference of novice drivers are about 2.5 s–3.5 s, general drivers are about 1.5s –2.5 s, and skilled drivers are about 1 s–1.5 s. This indicates that drivers with more driving experience usually make trajectory decisions after having a certain level of understanding of the environment.

After the driver makes a trajectory decision, they will obtain their expected vehicle motion trajectory, but a complete decision-making process also requires obtaining the steering instructions that need to be executed. According to the preview-following theory, drivers always try to keep the actual motion trajectory of the vehicle aligned with their expected trajectory while driving, so as to minimize driving errors^11^. Usually, the driver will set a preview point directly in front of the trajectory, and in order to make the vehicle trajectory coincide with the expected trajectory, the driver will try to keep the vehicle on the expected trajectory as much as possible.

Based on the driver’s preview characteristics and the assumption that the vehicle trajectory coincides with the expected trajectory after preview time, the steering action required by the driver to achieve the expected trajectory tracking can be derived based on the vehicle kinematics theory and the vehicle lateral acceleration gain. Therefore, it can be considered that the driver’s steering action decision is actually based on the deviation between their preview point and the expected trajectory, and the position of the preview point directly affects the steering action decision.

**Fig. 5:**
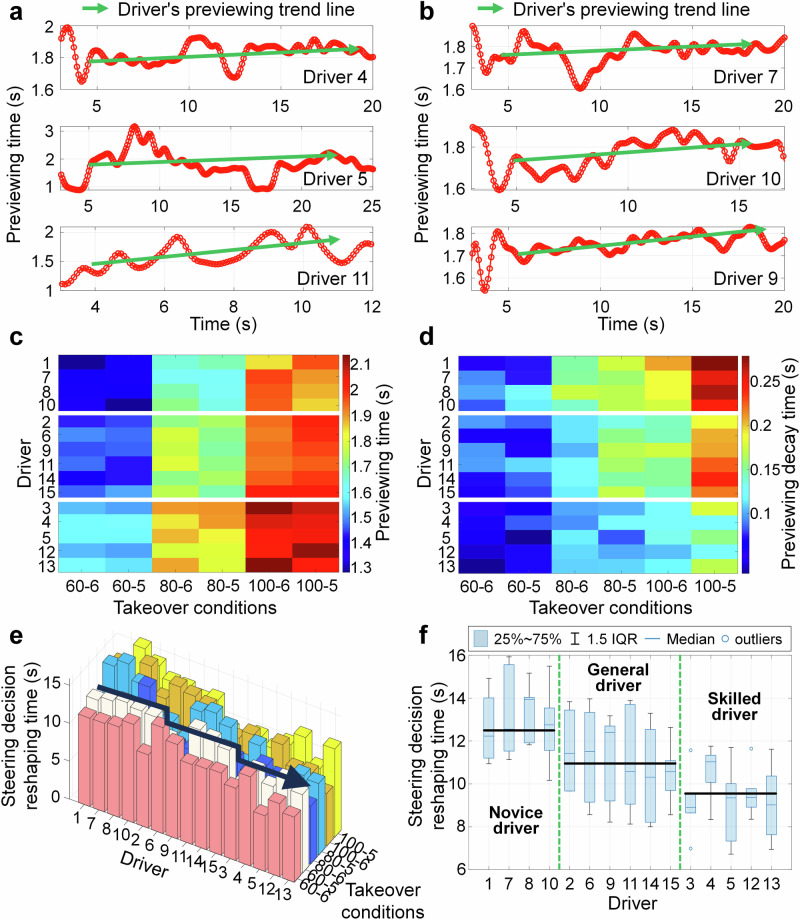
Comparison of preview time and steering decision reshaping time for different drivers. **a** Preview time of drivers 4, 5, and 11. **b** Preview time of drivers 7, 9, and 10. **c** Stable preview time of different drivers. **d** Preview decay time of different drivers. **e** 3-D histogram of steering decision reshaping time for different drivers. **f** Boxplot of steering decision reshaping time for different drivers.

Considering that the driver’s preview behavior is usually observation of distant roads, the vertical displacement of their line of sight in the scene can be used to calculate the preview distance. Then, based on the preview distance, the preview time is calculated to characterize the changes in its preview characteristics, and the decision characteristics of its turning action are analyzed. We select 2 novice drivers, 2 general drivers, and 2 skilled drivers as examples for analysis, as shown in Fig. 5a, b, the change curves of preview time for different drivers under the 80 km h^−1^ vehicle speed takeover are presented. It can be seen that the preview time of different drivers generally shows an upward trend, indicating that the preview characteristics of drivers are a progressive reshaping process, and indirectly indicating that the driver’s decision-making behavior towards steering actions is also progressively reshaped.

In addition, according to Fig. 5a, b, it can be seen that there are certain differences in the reshaping speed, stability value, and attenuation of drivers’ steering decisions, which are manifested in the different inclinations, starting and ending values of the green arrows in the figure. To maintain the progressive reshaping characteristics difference of steering action decision-making of different drivers under different operating conditions, we compare the preview time, decay amount, and steering decision reshaping time of different drivers under different working conditions and reserved takeover time, as shown in Fig. 5c, f.

From Fig. 5c, it can be seen that the takeover reservation time has a relatively small impact on the stable preview time, and the color distribution of different reservation times in the figure is relatively uniform. After the vehicle speed changes, the stable preview time of the driver increases, which is also in line with the existing research conclusion^34^. At a speed of 60 km h^−1^, the stable preview time for the driver is approximately between 1.3 s and 1.6 s, at 80 km h^−1^ it is approximately between 1.7 s and 1.9 s, and at 100 km h^−1^ it is between 1.85 s and 2.1 s. For driving proficiency, it can be seen from the chromatographic changes that as proficiency increases, the stable preview time of the driver also increases to some extent. According to Fig. 5 (d), the correlation between the preview decay time and vehicle speed for different drivers is higher than that of driving proficiency, and the preview decay time when the driver takes over the vehicle increases with the increase of vehicle speed. This indicates that the driver pays more attention to the nearby driving environment in the early takeover stage. For driving proficiency, an increase in driving proficiency helps to reduce the preview decay time during the takeover process, but the magnitude is small. This indicates that different drivers will prioritize environmental risks in the vicinity during emergency takeover, and gradually return to the normal preview state over time.

From Fig. 5e, f and Supplementary Tab. 4, it can be seen that the correlation between the reshaping time of steering decision and takeover conditions for different drivers is not obvious, but shows a strong correlation with driving proficiency, and as driving proficiency increases, the reshaping time of steering decision for drivers becomes shorter. The steering decision reshaping time for novice drivers is about 10.8s–15.7 s, general drivers are about 8.2s–13.7 s, and skilled drivers are about 7.2s–11.3 s. It can be seen that the reshaping time of the steering decision is longer than the completion time of the trajectory decision, mainly because the trajectory decision-making is a necessary decision for safety, while the steering decision reshaping is mainly a compensation decision made to better track the trajectory. So, the reshaping process of the driver’s decision-making characteristics refers more to the decision-making on the target trajectory, and the subsequent steering decision is a behavioral adjustment made by the driver under the premise of ensuring safe driving.

### Control behavior progressive reshaping mechanism of human drivers

The control behavior of human drivers mainly involves regulating the output torque of the neuromuscular system to the steering wheel of the vehicle based on the expected steering motion, so as to control the vehicle’s movement and track the expected trajectory and steering decision instructions^35^. Meanwhile, due to the fact that the driver’s torque control usually precedes their steering angle control, both show the same trend of change. To simplify the analysis, we consider the control state characteristics of the driver during the takeover process as the response characteristics of their neuromuscular system. In addition, considering that the driver’s steering control action usually starts from the decision point, this point can also be regarded as the starting point for reshaping the driver’s control behavior. Therefore, we analyze the driver’s neuromuscular response based on the collected EMG signals, and further dissect the progressive reshaping mechanism of the driver’s control behavior during the takeover process.

**Fig. 6:**
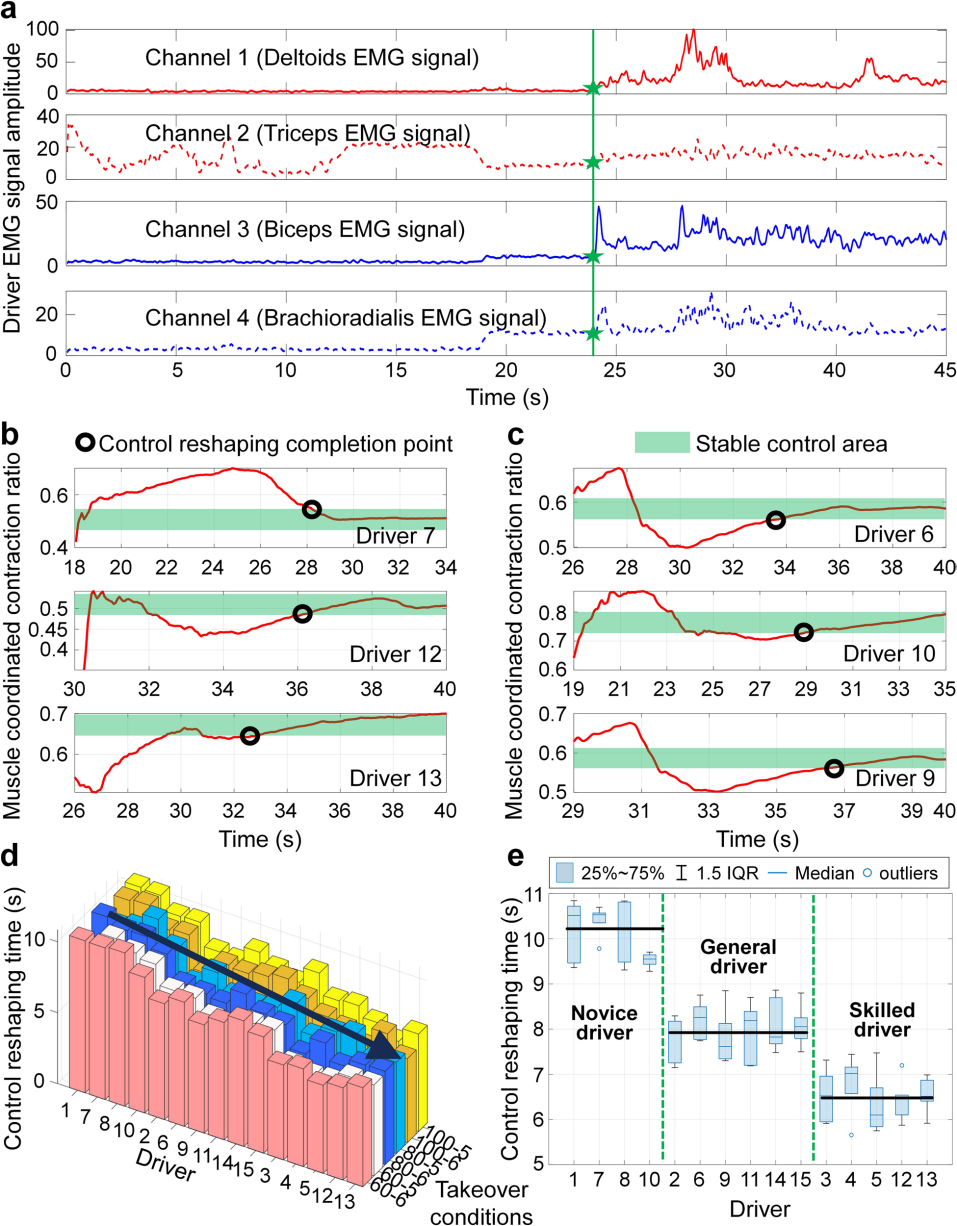
Muscle signals control the reshaping time of different drivers. **a** EMG signal envelope of different muscles of the driver. **b** Muscle coordinated contraction ratio of drivers 7, 12, and 13. **c** Muscle coordinated contraction ratio of drivers 6, 9, and 10. **d** 3-D histogram of control reshaping time for different drivers. **e** Boxplot of control reshaping time for different drivers.

As shown in Fig. 6a, the EMG signal envelopes of different channels for the driver are presented. According to the EMG signal envelopes of channel 2 and channel 4 in Fig. 6a, it can be seen that the driver has some muscle activity before the takeover mark, mainly due to the non-driving tasks engaged by the driver. After the takeover point, it can be seen that there are certain EMG signals in each channel, indicating that there is a coordinated contraction of the driver’s arm muscles during the takeover process. Muscle coordinated contraction refers to a phenomenon of muscle contraction involving both active and antagonistic muscles, aimed at achieving precise control of movement. The degree of muscle coordination of drivers is usually stable, but in some special situations, such as physical tension or discomfort in the external environment, the degree of muscle coordination may increase. At the same time, muscles increase muscle activity during coordinated contraction, thereby increasing the internal stiffness and damping of the arm. It can be considered that muscle-coordinated contraction is a process of muscle adaptive strengthening. Therefore, it is necessary to quantify the degree of muscle coordination contraction of drivers, analyze the changes in muscle activity of drivers during the takeover process, and clarify the mechanism of changes in driving characteristics during the takeover process. Usually, when characterizing the phenomenon of coordinated contraction between muscles, the muscle coordinated contraction ratio is used. It is defined as the envelope area of the EMG signal of the antagonistic muscle divided by the sum of the envelope areas of the EMG signals of the active muscle and the antagonistic muscle, which is mathematically expressed as:

1 $$CR(k)={\sum }_{i=1}^{k}A{n}_{M}(i)\cdot \Delta {T}_{E}/{\sum }_{i=1}^{k}[A{n}_{M}(i)+A{c}_{M}(i)]\cdot \Delta {T}_{E}$$

where *CR*(*k*) is the muscle coordinated contraction ratio of drivers at moment *k*, *Ac*_*M*_ and *An*_*M*_ are the EMG signal amplitudes of the active muscles and antagonistic muscles, respectively.

∆ *T*_*E*_ is the sampling period of the EMG signal of the driver’s arm muscles.

The existing research results indicate that the muscle coordinated contraction ratio of drivers should be moderate, not larger or smaller, the better^36^. Moderate muscle coordinated contractions can be beneficial in some cases, improving handling stability and accuracy. If the contraction is excessive, it will affect the driver’s handling performance. To analyze the muscle coordination and contraction characteristics of different drivers, 2 novice drivers, 2 general drivers, and 2 skilled drivers are selected as examples for analysis. The muscle coordination contraction ratio curves of different drivers are shown in Fig. 6b, c. It can be seen that the muscle coordination level of different drivers in the early stage of takeover will quickly climb to a certain level, mainly because the muscle activity of drivers in non-driving tasks is relatively small, while the muscle coordination contraction required for steering control is higher. Meanwhile, as the takeover process progresses, the degree of muscle coordinated contraction of the driver shows a process of increasing, oscillating, and then stabilizing. To mark the control reshaping completion point of drivers, we define the stage where the driver’s muscle coordination contraction ratio does not fluctuate by more than 10% as the stable stage, and define the range of muscle coordination contraction ratio variation during this stage as the driver’s stable control area.

According to Fig. 6b, c, there are differences in the steady-state values of muscle coordination contraction ratio among different drivers, mainly influenced by their physical condition and control habits. Individual differences are significant and not conducive to overall phenomenon analysis and observation. From the perspective of the overall trend of the driver’s muscle coordinated contraction ratio during the takeover process, the overall change is similar to the attenuated vibration motion of a damping structural system, and this characteristic can be described by the driver’s control reshaping time. Therefore, in order to further observe the differences in the characteristics of different drivers during the control process, we compare the reshaping time of different drivers under different working conditions and reserved takeover time, as shown in Fig. 6d, e.

From Fig. 6d, e and Supplementary Tab. 5, it can be seen that the correlation between the control reshaping time of different drivers and takeover conditions is not obvious, but shows a strong correlation with driving proficiency, manifested as the control reshaping time of drivers becomes shorter as driving proficiency increases. The control reshaping time for skilled drivers is about 5.8s–7.2 s, general drivers are about 7.2s–8.6 s, and novice drivers are about 9.3s–10.8 s. This process is mainly influenced by the driver’s usual driving experience and muscle sensitivity to driving. Generally, skilled drivers can quickly recover their level of vehicle control, while novice drivers, due to their lack of experience in handling emergency situations, are easily affected by tension and make excessive adjustments, resulting in longer recovery time for control.

### Cross-progressive reshaping mechanism of different driving behaviors

Based on the cognitive, decision, and control reshaping time of the driver during emergency takeover, it can be seen that the cognitive reshaping time period is usually 0–6 s, the decision reshaping time period is 2.5–15 s, and the control reshaping time period is generally 2.5–11 s. Therefore, the process of reshaping the driver’s cognitive, decision, and control behavior exhibits a series of parallel crossover characteristics, with the three interconnected on the timeline and reshaping in parallel within 2.5–6 s and 6–11 s, respectively. The cross-reshaping relationship diagram of different driver behaviors is shown in Supplementary Fig. S1. It can be seen from Supplementary Fig. S1 that after the emergency takeover, the autonomous driving system gradually exits the vehicle control, and the driver gradually takes over the vehicle control. During this process, the driver’s cognitive behavior begins to recover earliest, and then their decision and control behaviors recover in parallel with cognition.

When the driver’s cognition is completely reshaped, the driver’s decision and control behavior continue to be reshaped in parallel until the driver’s control behavior is completely reshaped and their decision behavior is sequentially reshaped to a normal level. These indicate that the different driving behaviors of drivers are not reshaped in chronological order, but rather have a serial and parallel cross-reshaping relationship with each other over time, which is also a key point that needs to be addressed in modeling driver driving behavior.

### Driving behavior progressive reshaping model testing

Considering that there is no relevant research on the proposed time-varying behavior model, a large amount of research is still in the stage of exploring and analyzing driver behavior, and a systematic modeling theory and method have not yet been formed. Therefore, we are unable to conduct a comparison and comparative analysis with SOTA methods in similar studies. This is because traditional driver models require a fixed driving route for tracking in advance, and the model parameters are fixed, which cannot describe the random, dynamic, and time-varying driving behavior characteristics of drivers in emergency takeover scenarios. However, for a completely new model, without comparison, its rationality and feasibility cannot be verified. Therefore, we referred to the discussion and research in the experimental verification section of the earliest driver modeling studies and found that the results of the earliest driver modeling studies were compared with real driver data, thereby proving that the model can reproduce the main behavioral characteristics of drivers^10,21^.

**Fig. 7:**
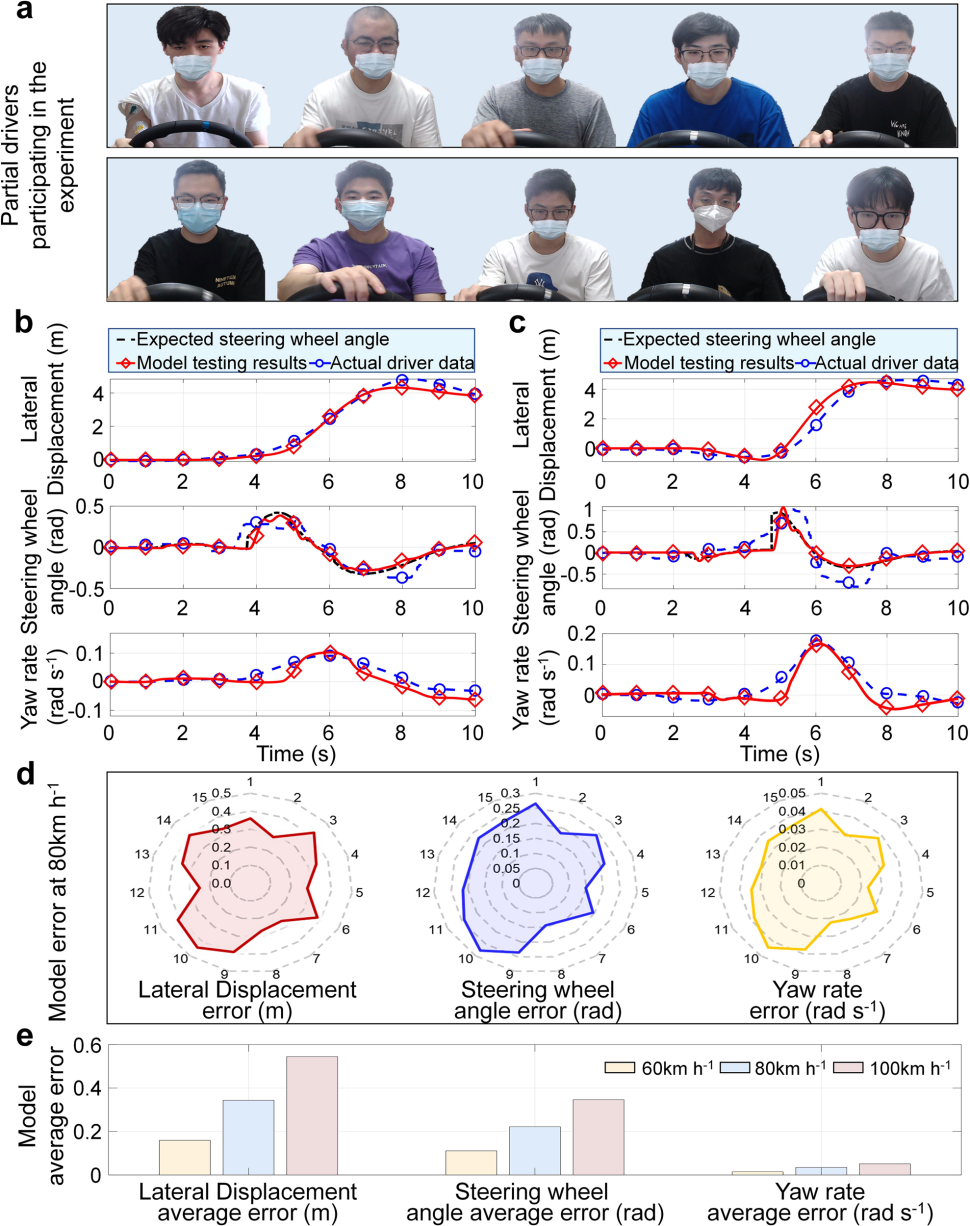
Verification results of the driver behaviors progressive reshaping model. **a** Partial driver. **b** Comparison of actual data and model for driver 4. **c** Comparison of actual data and model for driver 9. **d** Average error of vehicle status under different drivers at 80 km h^-1^. **e** The average error of vehicle status under different driving speeds for all drivers.

Therefore, in order to verify the rationality of the driver behavior progressive reshaping model, the actual driving data from different drivers is extracted, and some of the participating drivers are shown in Fig. 7a. Then, this paper identifies and extracts the takeover response parameters and physiological state parameters of different drivers, including state reshaping time, arm stiffness, damping, and moment of inertia. Finally, the actual parameters of different drivers are input into the driver behavior progressive reshaping model, and the driver model is used to replace the actual driver in the human-vehicle-road closed-loop system for emergency takeover experiments.

To verify the representational ability of the driver model on driver takeover performance, this paper extracts actual driving data of driver 4 and driver 9, and compares them with model validation data, as shown in Fig. 7b, c. It can be seen that the established driver model has good representation ability for the actual driving behavior of drivers 4 and 9. For the lateral displacement, the model and the actual driver have similar trajectories. For the steering wheel angle, the driver model has similar handling behavior to the actual driver in the same scenario. For the yaw rate, the driver model and the actual driving state of the vehicle have the same trend. These indicate that the proposed driver model can effectively express the driving behavior of drivers in emergency takeover scenarios. According to Fig. 7d, it can be seen that the actual driving data of different drivers and the lateral displacement error of the model are all within 0.45 m, the steering wheel angle error is within 0.3 rad, and the yaw rate error is within 0.045 rad s^−1^, indicating that the proposed model has good representation ability for the driving behavior of different drivers. According to Fig. 7e, as the emergency takeover speed increases, the driver model proposed will have a certain weakening ability to characterize the driver’s driving behavior; that is, the average errors of lateral displacement, steering wheel angle, and yaw rate will increase with the increase of vehicle speed. The average errors of the driver model in lateral displacement, steering wheel angle, and yaw rate at different vehicle speeds are 0.35 m, 0.22 rad, and 0.034 rad s^−1^, respectively. Compared with the average vehicle state under real driver control, the model accuracy in the three vehicle states is 89%, 88.3%, and 88.4%, respectively, with an average model accuracy of 88.57%. The results indicate that the overall error of the model is relatively small, indicating that the proposed driver model has good representation ability in different emergency takeover scenarios.

In addition, according to Fig. 7, it can be seen that there is a certain difference in the performance between the driver model established in this article and the real driver data, mainly due to the influence of model parameters. This article can continuously adjust the model parameters to make the model fit the real driver data, but unfortunately, it is difficult to determine the range of model parameters in a regular manner. Through comparative analysis, we have limited the range of the main parameters in the model, making it universal while minimizing errors, and enabling the model to reproduce the main time-varying driving behavior characteristics of drivers. The value range of *k*_*c*0_ in the model is 1-10. The parameters p1-p6 are mainly obtained by fitting the driver’s muscle coordinated contraction rate, with values ranging from 0.5–0.8, 0.06–0.2, 0.006–0.016, 0–1, 1–3, and 1–6, respectively. *t*_*c*0_ is the time for reshaping steering decisions, which can be taken from the experimental test data ranging from 6 to 15, depending on the scenario and driving proficiency. According to the comparison error between different driver models and their real driving data in Supplementary Tab. 6, it can be seen that the proposed model can not only reproduce the main takeover behavior characteristics of the driver, but also ensure that the lateral offset error is within 0.45 m, the steering wheel angle error is within 0.3 rad, and the yaw rate error is within 0.043 rad s^−1^, with high model accuracy and driving behavior reproduction ability.

**Fig. 8:**
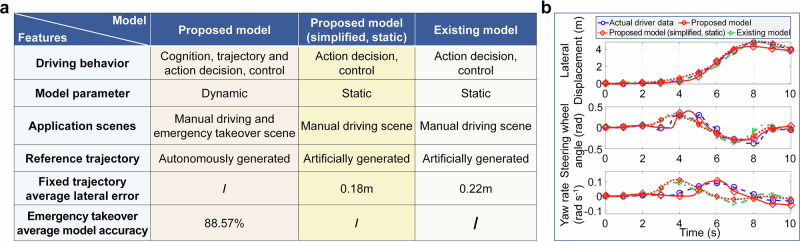
Qualitative comparison and partial quantitative comparison analysis results of driver models. **a** Qualitative comparison of driver models. **b** Partial quantitative comparison analysis results of driver models.

In addition, in order to further verify the progressiveness and rationality of the proposed driver model, this paper conducts qualitative and partial quantitative comparative analysis between the proposed driver model and the existing mainstream driver models^22,23^, as shown in Fig. 8a. It can be seen that the proposed driver model differs significantly from existing models in terms of model structure and application scenes. One is that the proposed model can comprehensively characterize the cognitive, decision, and control behavior characteristics of drivers, while existing models mainly focus on modeling driver action decision and control behaviors. Secondly, the proposed model is a dynamic and time-varying driver model, while the existing model is a static and parameter fixed driver model. The third is that the proposed model can autonomously cognize and generate target trajectories in different scenarios, while existing models must provide reference trajectories to simulate driver behavior. The fourth is that the proposed model can simplify the existing model architecture by fixing the parameters and discarding some modules, which can be used for manual and emergency takeover scenes, while the existing model can only be used for manual driving scenes. In view of this, it can be seen that the proposed model has greater advantages in driver behavior representation and application scenes.

Furthermore, in order to quantitatively compare with existing models, we will simplify the proposed model to make its representation range of driving behavior consistent with the existing model, and select fixed driver parameters as inputs for both models. Meanwhile, select the driving trajectory of Driver 4 as the reference trajectory. The comparison results are shown in Fig. 8a, b. It can be seen that the proposed simplified mode and the existing model can both achieve precise tracking of the reference trajectory. The lateral errors of the two are 0.18 m and 0.22 m, respectively, which indicates that the proposed model is slightly superior to the existing model in action decision and control behavior modeling. However, according to the steering wheel angle and yaw rate curves of the two models, it can be seen that due to the fixed reference trajectory, the driver model usually adopts a more ideal steering scheme to track the trajectory, which is significantly different from the steering behavior of the real driver during the takeover process. This phenomenon indicates that the existing static driver model is difficult to use to characterize the emergency takeover behavior of drivers. The proposed model not only characterizes the emergency takeover driving behavior of drivers but also achieves an accuracy of 88.57%, proving its superiority and rationality.

In summary, the established driver model, when using actual driver parameters, cannot only effectively characterize the time-varying progressive reshaping characteristics of different driver cognition, decision-making, and control behaviors, but also effectively express the changing patterns of driver control states. The results indicate that the established driver behavior progressive reshaping model has strong physical significance and rationality, and can effectively support the safe development and design of autonomous driving technology.

## Discussion

In this work, we built a hardware-in-the-loop experimental platform that can be used to conduct takeover experiments, and organized 15 drivers with different driving proficiency and gender to participate in the experiments. We simulated typical accident-prone takeover scenes in real driving environments by setting up takeover scenes at different vehicle speeds. By recording the visual and EMG data of different drivers, and vehicle status data during the takeover process, we analyzed in detail the time-varying characteristics of drivers’ cognition, decision, and control behavior. Based on the analysis results, we conclude that the behavior of drivers exhibits a progressive reshaping characteristic, and there is a temporal cross-correlation between them. Meanwhile, the progressive reshaping characteristics of drivers’ cognitive behavior can be observed and represented by their visual signals, while the progressive reshaping characteristics of decision-making behavior can be represented by changes in their preview behavior, and the progressive reshaping characteristics of control behavior can be represented by changes in their muscle state. In addition, based on the driver’s cognition, decision, and control reshaping time, it can be seen that with the takeover trigger moment as the origin of the timeline, the cognitive reshaping time period is usually 0–6 s, the decision reshaping time period is usually 2.5–15 s, and the control reshaping time period is usually 2.5–11 s. So, the reshaping process of driver’s cognition, decision, and control behavior exhibits a serial parallel crossover characteristic, with the three interconnected in the timeline and reshaping in parallel within 2.5–6 s and 6–11 s, respectively.

On this basis, we proposed a progressive reshaping model for driver behavior, which can describe the time-varying behavior characteristics during the driver takeover process based on the evolutionary mechanism of human driver behavior, and fully express the time-varying reshaping characteristics of different driver behaviors in series and parallel using time-varying model parameters. Finally, by comparing the driving data of real drivers with the driving data of the model, the feasibility and rationality of the constructed model were verified.

This study demonstrates that the driving behaviors of drivers have undergone significant changes in the context of autonomous driving, and traditional driver models can no longer describe these characteristics in the era of autonomous driving. The experimental research conducted in this article provides a new description of driver behavior in terms of principles and methods, which can effectively support the safe development of autonomous driving technology and have certain application value for the development of autonomous driving technology. Its value is mainly reflected in guiding the safety design of the autonomous driving system. For example, in the perception and recognition stage, when the system detects that it is about to enter a takeover scenario, it can combine the constructed model to evaluate the driver’s cognitive, decision, and control ability recovery level in real-time. In the model inference stage, the potential driving behavior and vehicle trajectory after taking over can be predicted based on the model, and the possibility of the driver successfully taking over control can be determined. In the control phase, the driver model and the vehicle model can be combined to form a man-machine cooperative driving system model, which can be used as the core and foundation of the predictive control of the autonomous driving system model.

In addition, compared to some studies with larger data scales, our study only covered 15 participants, which has certain limitations in terms of data size and comprehensiveness. For drivers, their driving proficiency, age, driving style, and body condition all have differentiated characteristics. We strive to ensure diversity in experience, gender, and driving habits among the participating drivers in the experiment. However, it is difficult to fully cover all the features of drivers with small sample sizes, which is also the main limitation of this article. To address this limitation, in future research, we will invite more drivers to participate in emergency takeover experiments, thereby expanding the data scale to enhance the generalizability of the research results. Meanwhile, we will refine the different characteristics and attributes of drivers to enhance the diversity and comprehensiveness of research.

## Methods

### Takeover experiments are designed for human drivers

**Fig. 9:**
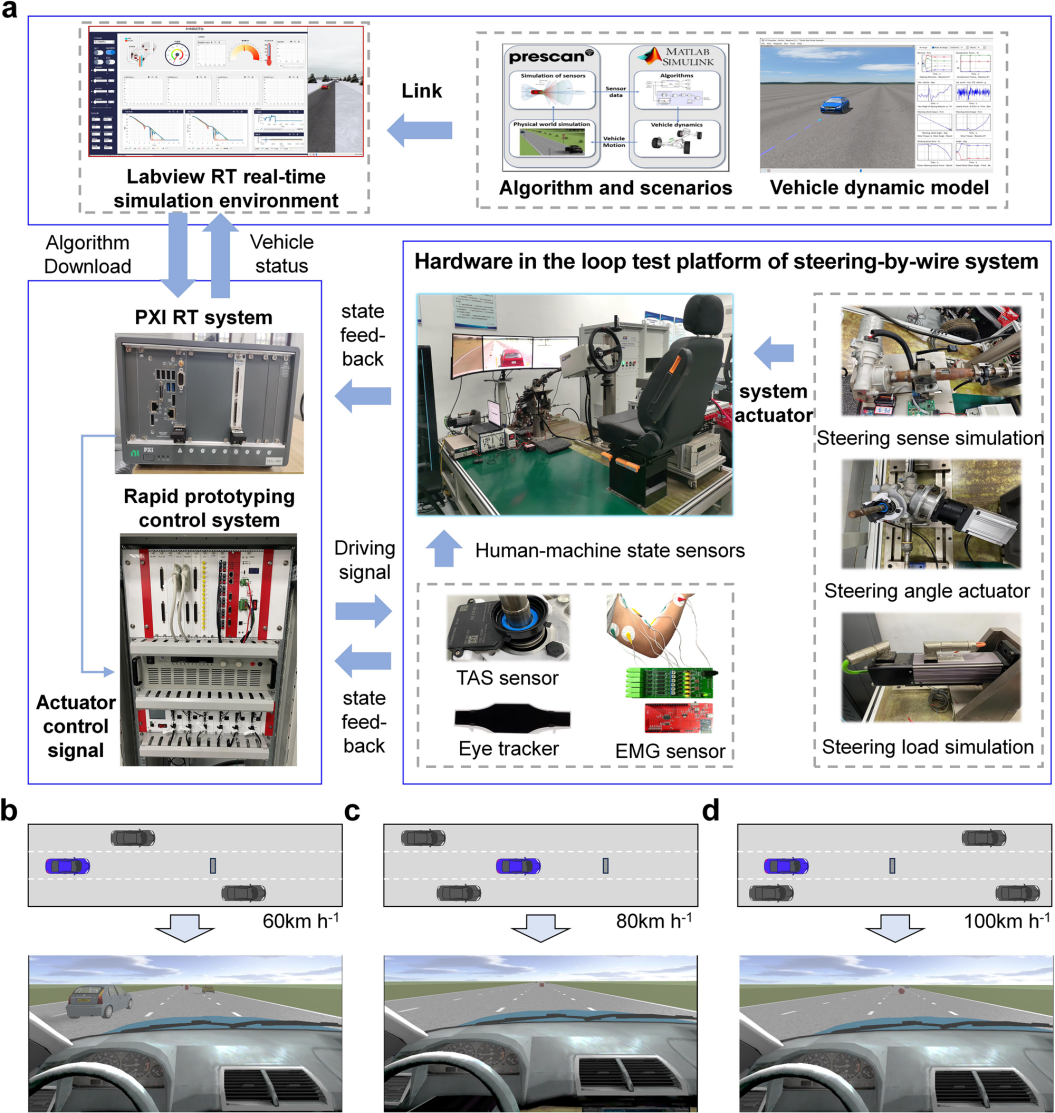
Emergency takeover experimental platform and scenarios. **a** The emergency takeover experimental platform. **b** Emergency takeover scenario at 60 km h^−1^. **c** Emergency takeover scenario at 80 km h^−1^. **d** Emergency takeover scenario at 100 km h^−1^.

To analyze the characteristic changes during the takeover process of drivers, it is necessary to obtain the real control and behavior data of drivers in the takeover scenario, as well as the vehicle operating status data. Therefore, we built a driver hardware in the loop experimental platform based on the actual vehicle hardware conditions, as shown in Fig. 9a.

Then, we design takeover experiments and record the experimental data of different drivers. To fully observe the driving characteristics under different takeover scenarios, we simulate different takeover scenarios by adjusting the takeover speed and the reserved time. Among them, the vehicle speeds are set to 60 km h^−1^, 80 km h^−1^, and 100 km h^−1^, respectively, and the reserved time is set to 4 s and 5 s, respectively. Before the emergency takeover test, the vehicle is driven by the autonomous driving system, and the speed is kept at the preset speed. The takeover reservation time is calculated using the collision warning time algorithm. When the collision warning time reaches the preset threshold, the takeover request is triggered. Meanwhile, we have complied with all relevant ethical regulations and obtained informed consent from all participants. Guidelines for study procedures were provided by Nanjing University of Aeronautics and Astronautics.

During the experiment, drivers are required to engage in non-driving tasks (playing games, chatting, watching videos, etc.). At this time, the vehicle is controlled by the autonomous driving system to drive at a constant speed. After running for a period of time, the vehicle will reach the preset scene. When the set takeover time is reached, the computer will use a beep signal to remind the driver to takeover. The takeover scene is shown in Fig. 9b–d.

As shown in Fig. 9b–d, the experimental road is a standard 3-lane road, where the ego vehicle travels at a constant speed in the middle lane. A raised obstacle is set up within a certain distance in front of the ego vehicle to simulate the main risk sources in takeover scenarios. The setting of road obstacles is selected according to the collision hazard level, and when the alarm is triggered, the obstacle position is marked for the driver. Meanwhile, in order to simulate real traffic operation scenarios, we set up four traffic vehicles to simulate actual traffic flow. The schematic diagram of the relative positions of the main vehicle and the environmental vehicles when taking over is shown in Fig. 9b–d (only the environmental vehicles within 300 m of the ego vehicle are drawn here). The vehicle speeds in different environments are set to 75, 80, 85, and 90 km h^−1^.

In addition, in order to reduce random and accidental results and achieve coverage of different types of drivers, we invite a total of 15 drivers with different driving experiences and genders who frequently drive vehicles to participate in takeover experiments, including 10 male drivers and 5 female drivers. Among them, there are 4 novice drivers, 6 general drivers, and 5 skilled drivers. Before the formal experiment, drivers are required to practice in the driving simulator for 10 minutes in advance to adapt to the simulator’s handling characteristics. After familiarizing themselves with the situation, drivers are required to conduct takeover experiments at different speeds and reserved takeover time according to the preset scenarios and working conditions. During this process, real-time data on the driver’s state is collected using eye-tracking devices, electromyography, and torque sensors. The vehicle status data is exported from the hardware-in-the-loop experimental platform.

### The progressive reshaping model of human driver driving behavior

**Fig. 10:**
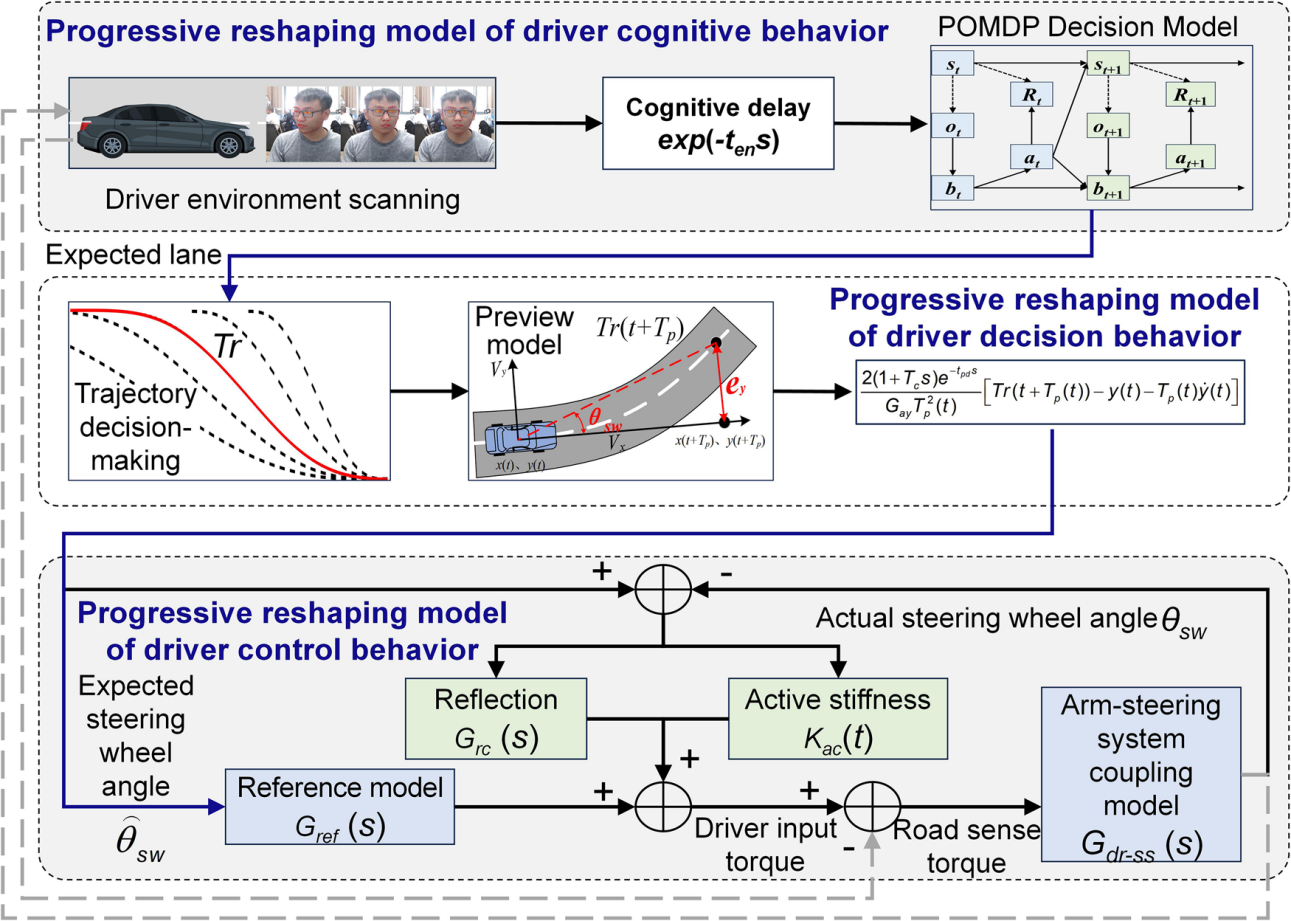
The progressive reshaping model of human driver driving behavior.

Experimental research has found that human drivers have progressive reshaping characteristics in cognition, decision-making, and handling. This article constructs a progressive reshaping model of human driver behavior based on the evolution mechanism of human driving behavior, as shown in Fig. 10.

### The progressive reshaping model of human driver cognitive behavior

The cognitive stage of drivers usually consists of three steps, namely environmental scanning, rapid decision-making, and decision adjustment. Considering that drivers usually recover their cognitive state during the environmental scanning phase, they do not engage in vehicle handling or decision behavior during this process. Therefore, this stage can be represented as a simple environmental information delay step *G*_*en*_, expressed as:

2 $${G}_{en}={e}^{-{t}_{en}s}$$

where *t*_*en*_ is the driver’s cognitive delay time, usually the time interval from the start of takeover to the decision point. According to the experiment analysis, its range is 0.5–1.3 s.

For the rapid decision-making stage, drivers are limited by environmental pressure due to incomplete environmental cognition, which can be considered as an empirical decision in a time-sensitive, information-incomplete, and highly uncertain environment. This characteristic is similar to the Partially Observable Markov Decision Process (POMDP)^37^. Due to the fact that unobservable characteristics generate more uncertainty in decision-making, POMDP quantifies these uncertain factors in the model to achieve decision-making in uncertain environments. Its expression can usually be represented by a six-tuple:

3 $${D}_{POMDP}=\{S,{A}_{de},T,R,O,OF,{B}_{de}\}$$

where *D*_*POMDP*_ is the driver’s rapid decision model, *S* is the state space, *T* is the state transition function, *A*_*de*_ is the action space, *R* is the return function, *O* is the observation space, *OF* is an observation model, and *B*_*de*_ is the belief state space.

Considering that both observable vehicles and the state of the ego vehicle can affect driver decisions, the state space is defined as the motion state of the ego vehicle and the motion state of environmental vehicles. Therefore, the state space at time *t* is represented as:

4 $${S}_{t}=[{s}_{ego},{s}_{1},{s}_{2},{{{\mathrm{..}}}}.,{s}_{{N}_{v}}],{s}_{i}=({X}_{t}^{i},{Y}_{t}^{i},{V}_{t}^{i},{\psi }_{t}^{i}),i=ego,1,2,{{{\mathrm{..}}}}.,{N}_{v}$$

where ${X}_{t}^{i}$,${Y}_{t}^{i}$, ${V}_{t}^{i}$ and ${\psi }_{t}^{i}$ represent the longitudinal displacement, lateral displacement, vehicle speed, and heading angle of vehicle *i* at time *t*, respectively. *N*_*v*_ is the number of environmental vehicles.

For the observation space, which is similar to the state space, drivers can usually perceive the position, speed, and heading information of the ego vehicle and surrounding vehicles. Therefore, the observation space of the model can be expressed as:

5 $${O}_{t}=[{o}_{ego},{o}_{1},{o}_{2},{{{\mathrm{..}}}}.,{o}_{{N}_{v}}],{o}_{i}=({X}_{ot}^{i},{Y}_{ot}^{i},{V}_{ot}^{i},{\psi }_{ot}^{i}),i=ego,1,2,{{{\mathrm{..}}}}.,{N}_{v}$$

For the observation model, it is primarily used to estimate the belief state distribution under the current state and driving actions. Since the driver possesses a certain level of cognitive ability before making the initial decision, it can be assumed that they have regained awareness of the vehicle’s operating state. Based on this assumption, it can be considered that the driver can accurately obtain the vehicle’s motion state. Therefore, the observation model in this paper is divided into two parts: the ego vehicle and surrounding vehicles, expressed as:

6 $$OF({o}_{t+1}|{s}_{t+1},{\alpha }_{t})=P({o}_{t+1}|{s}_{t+1},{\alpha }_{t})=P({o}_{t+1}^{ego}|{s}_{t+1}^{ego}){\prod }_{i=1}^{{N}_{v}}P({o}_{t+1}^{i}|{s}_{t+1}^{i})$$

For surrounding vehicles, since the driver has not yet fully regained cognitive awareness, it can be assumed that there is a certain level of uncertainty noise in their perception of the environment. Hence, the observation model of the surrounding vehicles can be defined as a probabilistic expression under the presence of noise:

7 $$\begin{array}{ll}P\left({X}_{t+1}^{i} \left| {s}_{t+1}^{i}\right.\right) = {N}\left({s}_{t+1}^{i},{\sigma }_{X}\right) & P\left({Y}_{t+1}^{i} \left| {s}_{t+1}^{i}\right.\right) = {N}\left({s}_{t+1}^{i},{\sigma }_{Y}\right) \\ P\left({V}_{t+1}^{i} \left| {s}_{t+1}^{i}\right.\right) = {N}\left({s}_{t+1}^{i},{\sigma }_{V}\right) & P\left({\psi }_{t+1}^{i} \left| {s}_{t+1}^{i}\right.\right)= {N}\left({s}_{t+1}^{i},{\sigma }_{\psi }\right)\end{array}$$

where *σ*_*X*_,*σ*_*Y*_,*σ*_*v*_ and *σ*_*ψ*_ represent the variance in longitudinal position, lateral position, speed, and heading angle, respectively. Their specific values can be determined by the variance of the actual driving data. Their values are 2507, 10.95, 20.06, and 8.53, respectively.

To express the characteristic of the driver’s cognitive progressive reshaping, it is assumed that when the driver fully regains cognition, they can accurately perceive the state of the environment. During the cognitive reshaping process, the driver’s perception ability of the environment gradually improves over time, reflected by the decreasing variance that approaches zero as time progresses. Thus, the driver’s observation uncertainty can be represented as a time-dependent decay function:

8 $${\sigma }_{j}={\sigma }_{j0}{e}^{-{k}_{co}(t-{t}_{en})},j=X,Y,V,\psi ;\,t={t}_{en}\sim {t}_{en}+{t}_{co}$$

where *σ*_*j0*_ represents the initial variance of the observation variable *j*. *k*_*co*_ is the variance decay coefficient used to adjust cognitive recovery speed, and its value range is 1-10. *t*_*co*_ is the decision-making reshaping time, which can be taken from the experimental test data ranging from 6 s to 15 s, depending on the scenario and driving proficiency.

For the state transition function, since the driver has complete knowledge of the ego vehicle’s motion state, the state transition function can be divided into the state transition of the ego vehicle and that of surrounding vehicles, expressed as:

9 $$T({s}_{t+1}|{s}_{t},{\alpha }_{t}) = 	 \, P({s}_{t+1}|{s}_{t},{\alpha }_{t})=P({s}_{t+1}^{ego}|{s}_{t}^{ego},{\alpha }_{t}^{ego}){\prod }_{i=1}^{{N}_{v}}P({s}_{t+1}^{i}|{s}_{t}^{i})\\ 	 P({s}_{t+1}^{ego}|{s}_{t}^{ego},{\alpha }_{t}^{ego})=1$$

Since the motion of the surrounding vehicles also conforms to the kinematic process, their state transition function can be defined as a kinematic model with uncertain noise errors:

10 $${s}_{t+1}^{i}=A{s}_{t}^{i}+{w}_{j}^{i},{w}_{j}^{i} : {{{\mathbf{N}}}}(0,{\sigma }_{j}^{i})$$

For surrounding vehicles, the probability distribution of different states is expressed as:

11 $$P({X}_{t+1}^{i}|{s}_{t}^{i}) = 	 \, {{{\mathbf{N}}}}({s}_{t}^{i},{\sigma }_{X})P({Y}_{t+1}^{i}|{s}_{t}^{i})={{{\mathbf{N}}}}({s}_{t}^{i},{\sigma }_{Y})\\ P({\psi }_{t+1}^{i}|{s}_{t}^{i}) = 	 \, {{{\mathbf{N}}}}({s}_{t}^{i},{\sigma }_{\psi })P({V}_{t+1}^{i}|{s}_{t}^{i})={{{\mathbf{N}}}}({s}_{t}^{i},{\sigma }_{V})$$

Similar to the observation model of surrounding vehicles, in order to express the driver’s cognitive progressive reshaping characteristic, this paper also represents the driver’s incomplete perception of the surrounding vehicle’s state as a time-decay function. Moreover, during the cognitive reshaping phase, it cannot be guaranteed that the driver will always obtain complete state information at every moment. Therefore, to maintain the Markov property of the model, the concept of belief state is introduced. The belief state primarily refers to the probability distribution of the state estimated by the driver based on historical information from the observation system, denoted as *b*(*s*). The set of all belief states forms the belief space *B*_*de*_, where all belief states satisfy:

12 $${\sum }_{s\in S}b(s)=1,b\in {B}_{de},0\le b(s)\le 1$$

In each step of the calculation process, the belief state can be described as the driver’s subjective probability. When the driver obtains new observations, the belief state can be updated using Bayes’ theorem. Thus, it can be seen that POMDP can be regarded as an MDP process in the belief space. The policy *π* can be viewed as a mapping from the belief space *B*_*de*_ to the action space *A*_*de*._ Therefore, when the belief state is *b*, the reward obtained by the driver when taking action *α* is:

13 $$r({b}_{t},{\alpha }_{t})={\sum }_{s\in S}R({s}_{t},{\alpha }_{t}){b}_{t}({s}_{t})$$

In addition, during the takeover process, due to the progressive reshaping of cognition and the changes in the urgency of the environment, drivers consider different goals when making decisions. For instance, in the early stages, when pressured by an urgent environment, the driver needs to make decisions within a limited time to ensure the safety of vehicle operation. At this time, the driver’s primary goal is safety. As their awareness of the global environment gradually recovers, the demand for driving comfort will gradually increase. Therefore, the reward function can be defined as a time-varying weighted multi-objective function:

14 $$R({s}_{t},{\alpha }_{t}) = 	 \, {w}_{s}{R}_{s}+\frac{{w}_{c}}{1+{e}^{a{d}_{1}-a{d}_{2}(t-{t}_{en})}}{R}_{c}\\ {R}_{s}({\alpha }_{t}) = 	 \, {w}_{TTC}\frac{{v}_{i}({\alpha }_{t})-{v}_{ego}}{{D}_{i-ego}({\alpha }_{t})}+{w}_{Th}\frac{{v}_{i}({\alpha }_{t})}{{D}_{i-ego}({\alpha }_{t})-L}$$

where *R*_*s*_ and *Rc* represent the safety objective and comfort objective, respectively. To simplify the calculation of *R*_*s*_, the current vehicle is translated to the corresponding lane based on the action *a*_*t*_ to compute the safety value for that lane. *w*_*s*_ and *w*_*c*_ are the weights for the safety and comfort objectives, respectively. *ad*_1_ and *ad*_2_ are adjustment factors. *w*_*TTC*_ and *w*_*Th*_ are the weights for the collision time and time headway. *D*_*i-ego*_ is the distance between the ego vehicle and surrounding vehicles, and *L* is the length of the ego vehicle.

Thus, the POMDP model can be transformed into the MDP model in the belief space to solve for its optimal value function Va* and the optimal policy:

15 $$V{a}^{\ast }({b}_{t}) = 	 \, {\min }_{\alpha \in A}\left\{{\sum }_{s\in S}{b}_{t}({s}_{t})R({s}_{t},{\alpha }_{t})+{\gamma }_{0}\left(\frac{1}{1+{e}^{a{d}_{1}-a{d}_{2}(t-{t}_{en})}}\right)\right. \\ 	 \left. \times {\sum }_{o\in O}OF({o}_{t}|{\alpha }_{t},{b}_{t})V{a}^{\ast }({b}_{t+1})\right\}\\ {\pi }^{\ast }({b}_{t}) = 	 \, {{\mbox{arg}}\,\min }_{\alpha \in A}\left\{{\sum }_{s\in S}{b}_{t}({s}_{t})R({s}_{t},{\alpha }_{t})+{\gamma }_{0}\left(\frac{1}{1+{e}^{a{d}_{1}-a{d}_{2}(t-{t}_{en})}}\right) \right. \\ 	 \left. \times {\sum }_{o\in O}OF({o}_{t}|{\alpha }_{t},{b}_{t})V{a}^{\ast }({b}_{t+1})\right\}$$

where *γ*_0_ represents the steady-state reward discount factor, and its value range is 0–1.

### The progressive reshaping model of human driver decision behavior

The progressive reshaping of driver decision-making behavior primarily involves the decision of the target trajectory and the steering action. For the target trajectory, since the lateral motion during the takeover process is critical for vehicle safety, this paper assumes that the vehicle speed remains constant during the takeover. Based on this information, a polynomial fitting method is used to obtain the final target trajectory, including the longitudinal and lateral trajectories.

When generating the longitudinal trajectory, let the initial time be t0 and the end time be te. A fifth-degree polynomial curve is used for fitting, expressed as:

16 $$\begin{array}{c}X(t)={a}_{0}+{a}_{1}t+{a}_{2}{t}^{2}+{a}_{3}{t}^{3}+{a}_{4}{t}^{4}\\ \left\{\begin{array}{l}X({t}_{0})={X}_{0} \hfill \\ {v}_{x}({t}_{0})={v}_{x}({t}_{e})={v}_{x0} \\ {a}_{x}({t}_{0})={a}_{x}({t}_{e})=0\end{array}\right.\end{array}$$

where *a*_*i*_ (*i* = 0,1,2,…,4) is the coefficient of the polynomial, *X*_0_ and *v*_*x*0_ are the longitudinal position and speed of the vehicle at time *t*_0_, respectively. *v*_*x*_ and *a*_*x*_ are the longitudinal speed and longitudinal acceleration of the vehicle.

For the lateral trajectory, unlike the longitudinal trajectory, the target lateral position is known. Therefore, a fifth-degree polynomial curve can also be used for fitting, expressed as:

17 $$\begin{array}{c}Y(t)={b}_{0}+{b}_{1}t+{b}_{2}{t}^{2}+{b}_{3}{t}^{3}+{b}_{4}{t}^{4}+b{t}^{5}\\ \left\{\begin{array}{l}Y({t}_{0})={Y}_{0},Y({t}_{e})={Y}_{e}({a}_{t})\\ {v}_{y}({t}_{0})={v}_{y}({t}_{e})=0\hfill\\ {a}_{y}({t}_{0})={a}_{y}({t}_{e})=0\hfill\end{array}\right.\end{array}$$

where *b*_*i*_ (*i* = 0,1,2,…,5) is the coefficient of the polynomial, *Y*_0_ and *Y*_*e*_ are the lateral position of the vehicle at time *t*_0_ and *t*_*e*_, respectively. *Y*_*e*_ represents the lateral position of the lane centerline corresponding to the decision action of the cognitive progressive reshaping model. *v*_*y*_ and *a*_*y*_ are the lateral speed and lateral acceleration of the vehicle.

Similar to the cognitive reshaping process, when deciding on the target trajectory, the driver is initially more focused on vehicle safety due to the urgency of the environment, gradually shifting their attention to the comfort objective in the later stages. To better align with this progressive reshaping characteristic of the driver’s decision-making, this paper plans five candidate trajectories at a granularity of 0.5 s. The trajectories are then filtered using the reward function *R*(*s*, *a*) from the cognitive progressive reshaping model as the objective function to simulate the driver’s decision-making behavior regarding the trajectories:

18 $$T{r}_{t}={{\mbox{arg}}\,\min }_{tr\in TR}\{tr[R({s}_{t},{a}_{t},t)]\}$$

where *Tr*_*t*_ is the target trajectory. When calculating the objective function for different candidate trajectories, it is assumed that the motion states of surrounding vehicles exhibit stable, continuous changes over a short period. *TR* is the set of candidate trajectories, and *tr* is a candidate trajectory.

After completing the trajectory decision, the driver generates control actions based on the target trajectory, which corresponds to the action decision. The driver’s action decision can be indirectly represented by the changes in the driver’s preview time, reflecting the progressive reshaping characteristic of the driver’s action decision-making process.

Typically, a driver’s behavior before executing steering actions can be divided into three processes: acquiring road information (which in this paper refers to obtaining the target trajectory), previewing road information, and generating desired steering angle commands. In this regard, the preview-following theory can describe the driver’s characteristic of minimizing the error between the expected preview path and the desired path^11^. Hence, this paper constructs a progressive reshaping model of driver action decision-making based on the preview-following theory. According to previous analyses, it is known that while the driver’s preview point may exhibit slight fluctuations during the action decision process, it generally remains close to a steady-state value. Thus, it can be assumed that the driver has a single-point preview characteristic.

According to the preview-following theory, the driver always aims to follow their desired trajectory *Tr* and establishes a preview point directly ahead of this trajectory while driving. The time required to reach this preview point at the current speed is referred to as the preview time. Based on kinematic principles, the lateral displacement of the vehicle after the preview time *T*_*p*_ can be expressed as:

19 $$Tr(t+{T}_{p})=y(t)+\dot{y}(t){T}_{p}+\frac{1}{2}\ddot{y}(t){T}_{p}^{2}$$

where $\ddot{y}$ is the lateral acceleration in the vehicle’s coordinate system.

Since the driver always aims for the vehicle’s position after the preview time to fall on the target trajectory, the lateral position after the preview time should minimize the error between the actual lateral position and the desired lateral position on the trajectory. Thus, it can be inferred that the driver’s ideal optimal lateral acceleration can be expressed as:

20 $${\ddot{y}}^{\ast }(t)=\frac{2}{{T}_{p}^{2}}[Tr(t+{T}_{p})-y(t)-{T}_{p}\dot{y}(t)]$$

Thus, the driver’s ideal optimal steering angle command can be expressed as:

21 $${\theta }_{sw}^{\ast }=\frac{2i{L}_{v}}{{V}^{2}{T}_{p}^{2}}[Tr(t+{T}_{p})-y(t)-{T}_{p}\dot{y}(t)]$$

where *i* is the transmission ratio from steering wheel angle to front wheel angle. *L*_*v*_ is the wheelbase of the vehicle.

Equation (21) can describe the driver’s expected steering behavior during low-speed steering. However, during normal road driving, the driver typically performs higher-speed or higher-frequency steering maneuvers. In this case, the relationship between the steering angle and lateral acceleration cannot be simplified to *V*^2^/*iL*_*v*_, but rather is expressed as:

22 $$\frac{\ddot{y}}{{\theta }_{sw}}(s)={G}_{{a}_{y}}\frac{1+{g}_{s1}s+{g}_{s2}{s}^{2}+{g}_{s3}{s}^{3}+{{\mathrm{.....}}}.}{1+{g}_{1}s+{g}_{2}{s}^{2}+{g}_{3}{s}^{3}+{{\mathrm{.....}}}.}$$

where *G*_*ay*_ is the lateral acceleration gain, which is the ratio of lateral acceleration to steering wheel angle when *s* = 0. *g*_*s*1_, *g*_*s*2_, *g*_*s*3_, *g*_1_, *g*_2_, *g*_3_ etc. are constants within the system.

By inverting the transfer function represented in Eq. (22) and integrating it into the system, the driver’s ideal steering angle can be obtained. Since it is difficult for the driver to perform such a complex computational process, a Taylor expansion is conducted, and this step is defined as the correction loop *C*(*s*), expressed as:

23 $$C(s)={C}_{0}(1+{T}_{c}s)$$

Based on Eq. (22), it can be observed that when *s* = 0, the system transfer function is a constant *G*_*ay*_ = 0.324. Thus, *C*_0_ = 1/*G*_*ay*_.

Additionally, considering that there is a certain neural delay in actual driving, this can be viewed as a pure time delay affecting the system. Therefore, the driver’s expected steering angle command can be expressed as:

24 $${{\theta }^{\frown {}}}_{sw}=\frac{2{C}_{0}}{{T}_{p}^{2}}[Tr(t+{T}_{p})-y(t)-{T}_{p}\dot{y}(t)](1+{T}_{c}s){e}^{-{t}_{nd}s}$$

where *t*_*nd*_ is the driver’s neural delay time, usually taken in 0.1–0.25 s.

Furthermore, during the takeover process, the driver’s preview point changes over time when making steering action decisions. To describe the progressive reshaping characteristics of the driver’s steering action decision-making, it is necessary to construct this time-varying property into the model. To simplify the modeling, this paper assumes that the driver’s preview time variation can be simplified as a linear time-varying characteristic, and thus the change in the driver’s preview characteristics can be expressed as:

25 $$\begin{array}{c}{T}_{p}={q}_{1}V+{q}_{2}{D}_{p}-{T}_{\Delta }+{T}_{\Delta }(t-{t}_{en})/{t}_{sm},t={t}_{en}\sim {t}_{en}+{t}_{sm}\\ {T}_{pe}=f(V,{D}_{p})={q}_{1}V+{q}_{2}{D}_{p}\iff {T}_{p}={T}_{pe}-{T}_{\Delta }+{T}_{\Delta }(t-{t}_{en})/{t}_{sm}\end{array}$$

where *T*_*pe*_ and *T*_*∆*_ are the driver’s steady-state preview time and the decay of preview time, respectively. *t*_*sm*_ is the steering decision reshaping time. *q*_1_ and *q*_2_ are constants used to characterize the rate of change of preview time with respect to vehicle speed *V* and the rate of change with respect to driving proficiency *D*_*p*_. Their value ranges are related to vehicle speed and driver proficiency, and in this article, their value ranges are 0.5–2.5 and 1–3, respectively.

By substituting the time-varying preview time model of the driver from Eq. (25) into the driver’s steering action decision model in Eq. (24), the driver’s steering action decision progressive reshaping model can be obtained.

### The progressive reshaping model of human driver control behavior

The changes in driver control characteristics are primarily reflected in the neuromuscular characteristics of their arms. Additionally, existing research has shown that neuromuscular characteristics play a crucial role in the steering control process of drivers^38^. Therefore, this paper constructs a progressive reshaping model of driver control behavior based on a neuromuscular model, including a reference model, a reflection control module, an active stiffness module, and an arm-steering dynamics model.

The arm-steering dynamics model simulates the process by which the driver’s input torque generates an angle. During normal driving, the driver’s arm and the steering system are highly coupled. To facilitate modeling, the coupled system of the driver and steering system is typically simplified to a spring-damper system. Thus, the arm-steering system coupling model can be expressed as:

26 $$({J}_{ss}+{J}_{dr}){\ddot{\theta }}_{sw}+({B}_{ss}+{B}_{dr}){\dot{\theta }}_{sw}+({K}_{dr}+{K}_{ss}){\theta }_{sw}={T}_{dr}-{T}_{rfa}$$

where *J*_*dr*_, *B*_*dr*_, *K*_*dr*_ and *J*_*ss*_, *B*_*ss*_, *K*_*ss*_ are the moment of inertia, damping, and stiffness of the driver’s arm and steering system, respectively. *T*_*dr*_ is the torque applied by the driver through their arm to the steering system. *T*_*rfa*_ is the road sense torque of the steering system. By converting the system into a transfer function that describes the relationship between the driver’s input torque and the steering wheel angle, it can be expressed as:

27 $${G}_{dr-ss}(s)=\frac{{\theta }_{sw}(s)}{{T}_{dr}(s)}=\frac{1}{({J}_{ss}+{J}_{dr}){s}^{2}+({B}_{ss}+{B}_{dr})s+({K}_{ss}+{K}_{dr})}$$

The reference model simulates the driver’s long-term adaptation process to the vehicle’s dynamic characteristics, reflecting their familiarity with the vehicle. In practice, the driver’s reference model may exhibit dynamic responses that are more akin to the characteristics of a second-order system. Moreover, the structure of a second-order system is simple and easy to identify^39^. So, this paper simplifies the reference model to a second-order system:

28 $${G}_{ref}(s)=\frac{{T}_{dr}(s)}{{\theta }_{sw}(s)}=\frac{r}{{r}_{2}{s}^{2}+{r}_{1}s+{r}_{0}}$$

where *r*, *r*_0_, *r*_1_, *r*_2_ are constants of the reference model.

The reflection control module primarily simulates the process from stimulus to muscle movement in the driver. Under reflection control, the stiffness and damping state of the driver’s muscles produce additional damping and stiffness values. For steering control, the reflection control module can use the deviation between the desired action and the actual action as the input. So, the transfer function of the reflection control can be expressed as:

29 $${G}_{rc}(s)={\omega }_{c}(s{B}_{rc}+{K}_{rc}){e}^{-{t}_{md}s}/s+{\omega }_{c}$$

where *ω*_*c*_ is the cutoff frequency of the reflection control module. *t*_*md*_ is the time delay generated by the motor neurons. *B*_*rc*_ and *K*_*rc*_ are the damping and stiffness of the reflection control module, respectively.

The active stiffness module primarily characterizes the increase in inherent muscle stiffness due to the co-contraction of the driver’s arm muscles. As the degree of co-contraction of the driver’s arm muscles increases, the internal stiffness and damping values of the driver’s arm muscles also increase. Considering that the co-contraction characteristics of the muscles mainly affect the driver’s active stiffness, this paper expresses the progressive reshaping characteristics of the driver’s control behavior in the active stiffness module. Based on previous analyses, the co-contraction rate of the driver’s arm muscles exhibits damping oscillation characteristics. Hence, a damping oscillation curve can be used to fit this characteristic of the driver:

30 $$CR={p}_{1}+{p}_{2}\times {e}^{-{p}_{3}\times {(t-{p}_{4})}^{3}}\times \,\sin ({p}_{5}\times (t-{p}_{4})+{p}_{6})$$

where *p*_1_, *p*_2_, *p*_3_, *p*_4_, *p*_5_, and *p*_6_ are parameters of the damping oscillation curve, which are fixed constants. Among them, *p*_1_ is the initial muscle state at the start of the takeover, *p*_2_ is the adjustment coefficient for the oscillation amplitude, *p*_3_ is the damping coefficient, *p*_5_ denotes the oscillation frequency, and *p*_6_ is the initial phase offset. The parameters p1-p6 are mainly obtained by fitting the driver’s muscle coordinated contraction rate, with values ranging from 0.5–0.8, 0.06–0.2, 0.006–0.016, 0–1, 1–3, and 1–6, respectively.

For active stiffness modeling, it can be used to express the time-varying characteristics of the driver’s arm muscle active stiffness during the takeover process, expressed as:

31 $${K}_{ac} = 	 \, {K}_{ac0}\wedge [{k}_{CR}(CR/C{R}_{e})] \\ = 	 \, {K}_{ac0}\wedge \left[{k}_{CR}\frac{{p}_{1}+{p}_{2}\times {e}^{-{p}_{3}\times {(t-{p}_{4})}^{3}}\times sin({p}_{5}\times (t-{p}_{4})+{p}_{6})}{C{R}_{e}}\right]$$

where *K*_*ac*0_ is the active stiffness of the driver’s muscles during normal driving, *k*_*CR*_ is the adjustment coefficient, and *CR*_*e*_ is the steady-state value of the driver’s arm muscles co-contraction rate.

### Ethics declaration

We have complied with all relevant ethical regulations and obtained informed consent from all participants. Guidelines for study procedures were provided by Nanjing University of Aeronautics and Astronautics. We have received ethical approval from Nanjing University of Aeronautics and Astronautics review board.

## Supplementary information

Supplementary Information

## Data Availability

The data that support the findings of this study are available from the corresponding authors upon reasonable request.

## Code availability

The data that support the findings of this study are available from the corresponding authors upon reasonable request.
