# Supplementary material for: Evolution mechanism and progressive reshaping model of driving behaviors when humans take over intelligent vehicles
Source: Commun Eng. 2025 Oct 31;4:181. doi: 10.1038/s44172-025-00510-6 (PMC12579248; doi:10.1038/s44172-025-00510-6)
Supplement: Supplementary file 1 — Supplementary Information [file 44172_2025_510_MOESM1_ESM.pdf]

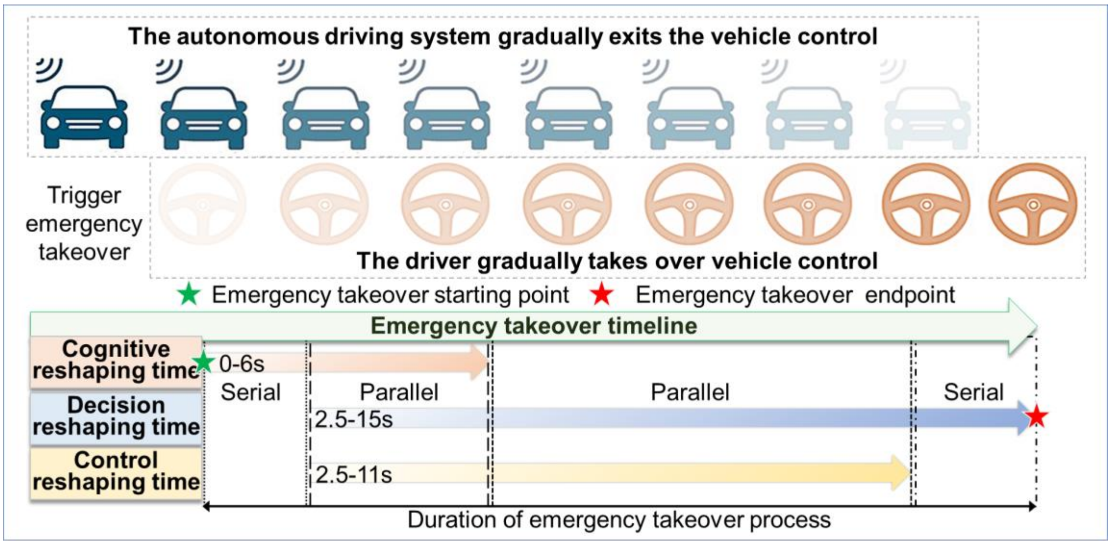

Supplementary Fig. S1 Cross progressive reshaping mechanism of different driving behaviors.

Supplementary Tab. 1 Standard deviation of steering data for different drivers during different takeover periods

	Driver1			Driver3			Driver9		
Takeover period (s)	0-3s	3-6s	6-9s	0-3s	3-6s	6-9s	0-3s	3-6s	6-9s
Steering wheel angle standard deviation (deg)	20.3	24.2	11.2	16.1	59.6	27.3	29.9	51.9	19.5
Lateral displacement standard deviation (m)	0.24	2.14	0.44	0.43	1.44	0.58	0.64	1.23	0.37

Supplementary Tab. 2 Cognitive reshaping time of different drivers in different emergency takeover scenarios (5s/6s represent the reserved takeover time)

Unit: (s)	60 km h^{-1}_6s	60 km h^{-1}_5s	80 km h^{-1}_6s	80 km h^{-1}_5s	100 km h^{-1}_6s	100 km h^{-1}_5s
Driver1	5.34	5.28	5.96	5.84	6.7	6.59
Driver7	5.26	5.19	6.04	5.95	6.67	6.54
Driver8	5.41	5.36	5.85	5.73	6.6	6.45
Driver10	5.24	5.22	5.91	5.76	6.75	6.54
Driver2	5.07	4.98	5.68	5.57	6.42	6.18
Driver6	4.95	4.84	5.57	5.47	6.38	6.27
Driver9	5.13	4.98	5.62	5.44	6.58	6.33
Driver11	5.09	4.88	5.51	5.37	6.64	6.63
Driver14	5.04	4.9	5.76	5.67	6.47	6.41
Driver15	4.99	4.92	5.69	5.58	6.33	6.13
Driver3	4.68	4.65	5.34	5.29	6.19	6
Driver4	4.48	4.35	5.24	5.13	6.02	5.87
Driver5	4.76	4.6	5.49	5.35	5.9	5.86
Driver12	4.45	4.22	5.35	5.32	6.19	5.95
Driver13	4.56	4.5	5.24	5.12	6.3	6.14

Supplementary Tab. 3 Decision point time of different drivers in different emergency takeover scenarios

Unit: (s)	60 km h^{-1}_6s	60 km h^{-1}_5s	80 km h^{-1}_6s	80 km h^{-1}_5s	100 km h^{-1}_6s	100 km h^{-1}_5s
Driver1	2.81	2.63	3.06	3.05	3.61	3.54
Driver7	2.69	2.61	3.26	3.12	3.65	3.65
Driver8	2.8	2.68	3.11	2.86	3.61	3.34
Driver10	2.57	2.66	3.01	3.01	3.55	3.49
Driver2	2.72	2.71	3.19	3.02	3.65	3.57
Driver6	2.72	2.63	3.08	2.94	3.66	3.6
Driver9	2.8	2.59	3.1	3.15	3.71	3.65
Driver11	2.76	2.56	3.01	2.94	3.78	3.72
Driver14	2.65	2.61	3.28	3.25	3.76	3.62
Driver15	2.61	2.52	3.22	2.96	3.52	3.4
Driver3	2.63	2.48	3	2.9	3.66	3.59
Driver4	2.53	2.33	3.02	2.97	3.44	3.36
Driver5	2.68	2.52	3.21	3.06	3.47	3.37
Driver12	2.56	2.35	3.14	3.02	3.62	3.54
Driver13	2.54	2.52	3.04	2.85	3.66	3.51

Supplementary Tab. 4 steering decision reshaping time of different drivers in different emergency takeover scenarios

Unit: (s)	60 km h^{-1}_6s	60 km h^{-1}_5s	80 km h^{-1}_6s	80 km h^{-1}_5s	100 km h^{-1}_6s	100 km h^{-1}_5s
Driver1	14.16	12.99	11.84	12.19	14.89	11.51
Driver7	15.99	12.66	13.77	11.67	10.05	14.46
Driver8	11.25	11.7	15.04	12.89	10.53	15.67
Driver10	12.79	10.17	15.74	15.98	14.08	15.8
Driver2	13.86	8.69	12.06	13.85	13.96	8.99
Driver6	8.79	13.03	10.31	12.07	11.62	12.28
Driver9	13.68	12.08	13.29	11.93	12.43	8.34
Driver11	8.49	8.31	9.56	13.31	12.29	13.3
Driver14	11.03	11.11	8.34	13.63	8.79	9.97
Driver15	10.27	13.38	12.95	9.64	9.05	13.63
Driver3	10.11	8.62	6.33	8.58	8.78	9
Driver4	7.45	10.44	6.44	11.44	6.58	11.54
Driver5	8.78	6.97	11.16	7.99	6.05	9.85
Driver12	8.97	9.99	7.14	9.36	11.56	10.73
Driver13	10.71	6.38	11.02	11.47	6.31	6.48

Supplementary Tab. 5 control reshaping time of different drivers in different emergency takeover scenarios

Unit: (s)	60 km h^{-1}_6s	60 km h^{-1}_5s	80 km h^{-1}_6s	80 km h^{-1}_5s	100 km h^{-1}_6s	100 km h^{-1}_5s
Driver1	10.35	9.9	10.06	10.54	9.85	10.33
Driver7	10.12	10.99	10.6	10.96	9.89	9.29
Driver8	10.1	9.46	9.65	9.82	10.18	9.08
Driver10	10.27	9.26	10.34	9.58	9.67	9.76
Driver2	8.54	7.42	8.8	8.82	8.08	8.02
Driver6	7.68	7.65	8.53	8.33	7.82	7.32
Driver9	8.32	7.28	8.31	8.14	7.27	7.57
Driver11	8.16	7.92	8.51	7.65	8.57	7.61
Driver14	7.45	8.48	8.74	7.11	8.55	8.88
Driver15	7.89	8.36	8.8	7.21	7.47	7.78
Driver3	6.5	5.8	6.32	6.69	5.83	6.9
Driver4	5.85	6.39	7.15	6.09	6.29	7.46
Driver5	6.43	5.87	6.48	6.15	7.32	6.4
Driver12	6.95	6.13	5.8	5.58	5.54	5.7
Driver13	5.82	6.07	5.74	6.07	6.73	6.81

Supplementary Tab. 6 Average error of vehicle status under different drivers' driving conditions at 80km h^{-1}

	Lateral Displacement Error (m)	Steering wheel angle error (rad)	Yaw rate error (rad s^{-1})
Driver1	0.36	0.25	0.04
Driver2	0.28	0.18	0.03
Driver3	0.42	0.28	0.04
Driver4	0.34	0.18	0.03
Driver5	0.28	0.2	0.03
Driver6	0.38	0.22	0.04
Driver7	0.26	0.16	0.02
Driver8	0.27	0.18	0.03
Driver9	0.39	0.24	0.04
Driver10	0.44	0.30	0.043
Driver11	0.41	0.29	0.04
Driver12	0.25	0.15	0.02
Driver13	0.35	0.18	0.03
Driver14	0.4	0.28	0.04
Driver15	0.33	0.26	0.04
